# Smart 3D Printed Hydrogel Skin Wound Bandages: A Review

**DOI:** 10.3390/polym14051012

**Published:** 2022-03-03

**Authors:** Filmon Tsegay, Mohamed Elsherif, Haider Butt

**Affiliations:** Department of Mechanical Engineering, Khalifa University, Abu Dhabi P.O. Box 127788, United Arab Emirates; mohamed.elsherif@ku.ac.ae

**Keywords:** wound dressings, 3D printing, bioprinting, drug delivery, sensor-integrated bandages

## Abstract

Wounds are a major health concern affecting the lives of millions of people. Some wounds may pass a threshold diameter to become unrecoverable by themselves. These wounds become chronic and may even lead to mortality. Recently, 3D printing technology, in association with biocompatible hydrogels, has emerged as a promising platform for developing smart wound dressings, overcoming several challenges. 3D printed wound dressings can be loaded with a variety of items, such as antibiotics, antibacterial nanoparticles, and other drugs that can accelerate wound healing rate. 3D printing is computerized, allowing each level of the printed part to be fully controlled in situ to produce the dressings desired. In this review, recent developments in hydrogel-based wound dressings made using 3D printing are covered. The most common biosensors integrated with 3D printed hydrogels for wound dressing applications are comprehensively discussed. Fundamental challenges for 3D printing and future prospects are highlighted. Additionally, some related nanomaterial-based hydrogels are recommended for future consideration.

## 1. Introduction

It is well-known that the human skin can regenerate naturally to overcome bruises and wounds. There are 20 million patients worldwide who suffer from chronic wounds, costing healthcare systems about USD 20 billion [1,2]. Skin wounds present the highest burden in the United States. In 2018, the estimated investment in treating acute and chronic wounds, was in the range of USD 28.1–96.8 billion [3]. Wound management has been shown to be a considerable burden in the United Kingdom too. Studies indicate that the amount spent on wound treatments is approximately 4% of annual public expenditure [4]. High expenses for treating wounds are associated with diabetic foot ulcers and outpatient wound care compared with inpatients [3]. The continued threat of diabetes and obesity means that chronic wounds present substantial clinical, social, and economic challenges worldwide [3]. Traditional skin wound dressings are often used as primary or secondary dressings to shield a skin wound from contamination. Common types of traditional wound dressings include bandages, body netting, cohesive wraps, composite dressings, impregnated gauze, non-adherent dressings, leaves, cobwebs, and honey [5]. One of the main drawbacks of traditional dressings is that they become attached to newly grown granulations and cause pain when removed [6]. There are significant differences between traditional and modern wound dressings. Modern wound dressings are multifunctional, provide physical protection, maintain the moisture content of the wound microenvironment, and accelerate the healing process by improving the wound healing rate. On the other side, traditional dressings cover the wound and absorb exudates only [7,8]. The type of wound dressing is selected based on the wound type, depth, body part location, and the extent of the wound [9]. Heyer et al. reported a comparison between traditional/conventional and advanced/modern wound dressings [10]. In a meta-analysis of 287 controlled and uncontrolled trials, the mean odds ratio for chronic wound healing was found to be 1.52, favoring advanced over traditional wound treatments in 65 controlled trials [10]. In experimental studies, an effective reduction in chronic infection was observed with advanced/modern wound dressings.

**Figure 1:**
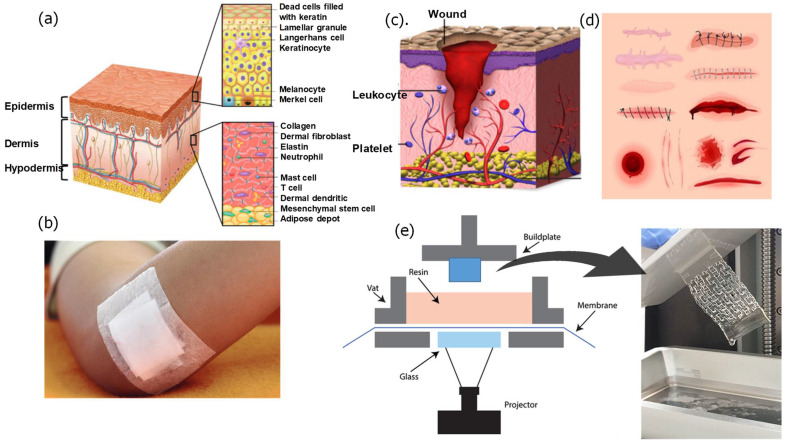
(**a**) Skin structure, (**b**) conventional wound dressing, (**c**) wound structure, (**d**) types of wounds and curing system, (**e**) VAT polymerization 3D printing system, and the produced hydrogel wound dressing.

The emergence of modern multifunctional wound dressings arose because of a great medical need, as some wounds pass a threshold diameter such that they cannot recover by themselves. Furthermore, in some patients, the wound becomes chronically impaired, and can lead to mortality [11]. Currently, the best methods for wound treatment are referred to as the ‘gold standard’. They include split and full-thickness skin grafts, skin flaps, skin expansion techniques, and dermal substitutes (Figure 1) [12,13,14]. The primary challenges associated with these techniques are the shortage of donor sites, and the hypertrophic scars or keloids arising that can lead to dysfunction or psychosocial problems [15,16]. In addition to high costs, the availability of techniques worldwide is another issue that requires attention. Therefore, there is a great need to devise new solutions to address these challenges. As mentioned, when conventional wound dressings are placed on the wound, they absorb the wound exudates and dry out, peeling off some tissues around the wound site whenever they are taken-off, resulting in further infection. Other side effects of traditional wound dressings include low oxygen permeability, non-biomimicry, and difficulty in loading with adequate levels of drugs. Tissue regeneration engineering has produced skin substitute techniques that offer promising alternatives to overcome these limitations. The tissue engineering field has produced a heterogeneous range of both temporary and permanent wound dressings [17]. However, skin biomimicry and mechanical strength are still challenges.

Promising features of 3D printing technology, in association with biocompatible hydrogels, may overcome the identified challenges for wound dressing. 3D printing technology allows for subtle micro-component design that can regulate the delivery of different biologically functional agents. During printing procedures, the hydrogel can be loaded with various items, such as antibacterial nanoparticles and other biological substances. The main advantages of usage 3D printing technique in manufacturing hydrogel wound patches are high reliability and cost-effectiveness. Conventional manufacturing techniques, including molding, casting, and forming and machining, are good for mass production, but they are not suitable for complex and multi-material designs.

Several review articles have considered 3D printed hydrogels used either for bone and tissue engineering or for medical applications in general. Relevant reviews from 2014 to date are summarized in this section. M.L. Pita-López et al. reported progress in the fabrication of physically cross-linked chitosan hydrogels for applications in tissue engineering [18]. The review highlighted the 3D printing techniques used for developing the chitosan hydrogel. R. Rodríguez-Rodríguez et al. reviewed advances in fabrication of composite hydrogels used for biomedical applications [19]. The applications included wound caring, drug delivery, and tissue engineering; however, 3D printing methods were not in the scope of the review. R. Pugliese et al. highlighted recent advances in the field of 3D printing hydrogels and their biomedical applications, focusing on analyzing the different hydrogels and bio-inks based on their printability properties, cost-effectiveness, degradation rate, and biocompatibility [20]. J. Li et al. reported a review which focused on hydrogel designs, and the development of hydrogel-bio-ink materials for 3D printing [21]. Stereolithography printing and inkjet printing techniques were covered. Several applications of 3D printed materials were highlighted, including regenerative medicine, tissue engineering, drug screening, and wearable electronics. M. Rajabi et al. summarized recent advances in chitosan-based hydrogels developed using 3D printing for biomedical applications [22]. The review provided an overview of the physical and chemical approaches used for synthesizing chitosan-based hydrogels as a 3D printable ink. S. Bakhtiari et al. considered nanocomposite hydrogels fabricated using 3D printing, focusing on the properties of the printed materials and their biomedical applications [23]. A. Zamboulis et al. reviewed recent advances in 3D printing of polysaccharides, paying attention to applications in drug delivery [24]. The review covered cellulose and cellulose derivatives, chitosan and sodium alginate which were printed using fused deposition modeling and extrusion-based printing. S. Agarwala et al. reviewed electrically conductive hydrogels, concentrating on composite hydrogels and their synthesis routes [25]. The fabrication, design, and components of conductive hydrogels were highlighted, and 3D printing, as an advanced approach for designing, functioning, and printing conductive polymers, was discussed. The review aim was to assist readers in selecting the most suitable methodology for designing desired composite conductive hydrogels. S.S Athukorala et al. reviewed electrically conductive hydrogels manufactured using 3D printing [26]. The review provided an overview of state-of-the-art 3D printed conductive polymers, including polythiophene, polyaniline, and polypyrrole. The review provided insights into the electric conductivity mechanisms and the design considerations for tunable physiochemical properties. In addition, recent advances in 3D printable bio-inks and their practical applications, were discussed. R.C. Advincula et al. presented an overview of progress in the development of 3D printed hydrogels used for tissue engineering applications [27]. The scope of the review included natural, synthetic, and nanocomposite polymers and their design considerations for 3D printing. A.I. Pérez-Sanpablo et al. presented cutting edge developments in 3D printing technology and their applications in healthcare [28]. Challenges related to 3D printing applications in healthcare were addressed, as well as consideration of the advantages and disadvantages of emerging 3D printing technology. M. Topuz et al. reported a review of the hydrogels used in bioprinting, including both natural and synthetic hydrogels [29]. G.M. Paul reported recent developments in the application of 3D printing in medicine [30]. The review evaluated the limitations of the 3D printing in medical applications. F. Fayyazbakhsh et al. provided an overview of 3D bioprinted substitutes [31]. The review addressed the use of bioprinting compositions and three types of 3D printing mechanisms, i.e., inkjet, microextrusion, and laser-assisted bioprinters. R.S de Oliveira et al. presented advances in 3D printing for developing pharmaceutical and biomedical products for drug delivery, skin applications, and plain dressings [32]. The study discussed composite, natural and synthetic hydrogels. W. Sun et al. reviewed 3D printing technology used for printing hydrogel actuators [33]. The actuation mechanisms, including pneumatic, hydraulic, ionic, dehydration-rehydration, and cell-power actuation, were addressed. Applications of hydrogel actuators in micro-swimmers, wearable devices, and origami structures, were discussed. N. Li et al. reviewed 3D printing technology used for printing biopolymers, i.e., polysaccharides and proteins [34]. The most commonly used printing mechanisms, including inkjet and extrusion-based printing, stereolithography, selective laser sintering, and binder jetting, were discussed. Applications of printed polysaccharides and proteins in food and biomedical fields were covered. N. Beheshtizadeh et al., reviewed 3D bioprinting applications in human tissues, particularly for bone scaffolds based on hydroxyapatite and biphasic calcium phosphate [35]. S. Derakhshanfar et al., described recent advances in bio-inks and bioprinting methods [36]. The review concentrated on the diverse types of crosslinking methods used in bio-inks, and the challenges related to 3D bioprinting for applications in medical sciences. A. Bauermeister et al. summarized ongoing research on 3D printing for applications in reconstructive surgery [37]. M. Askari et al. considered hydrogel–based bioprinted scaffolds, including skin, bone, cartilage, vascular, neural, and muscular scaffolds [38]. The study focused on 3D bioprinting methods, including multi-dispenser, coaxial, and hybrid bioprinting. G. Souvik et al. provided an overview of the basic principles of 3D bioprinting and the diverse 3D printing mechanisms [39]. The basic components of bio-inks used in skin bioprinting were discussed. The study analyzed the skin constructs developed by 3D bioprinting. S. Gupta et al. introduced the conventional and 3D printing technologies used for manufacturing biomaterials for biomedical applications [40]. Primary attention in the study was paid to FDM-based printing techniques. P. He et al. summarized strategies for 3D bioprinting and recent advances in the bioprinting of skin constructs [41]. The study highlighted the challenges of 3D bioprinting for skin regeneration. H. Li et al. evaluated the significant potential of bio-inks and 3D printing developed constructs [42]. Properties of bio-inks, including degradation rate, and structural, biological, interfacial, and rheological features were summarized. In addition, the most commonly used hydrogels for 3D bioprinting were addressed. X. Li et al. reviewed advances in the development of biopolymer materials, including both natural and synthetic biopolymers [43]. 3D printing technology, as an advanced production approach for biopolymers, was briefly covered. M. Sahranavard et al. presented an overview of 3D printing mechanisms used for developing chitosan scaffolds and their applications in the biomedical field [44]. Recent progress in 3D printed chitosan matrices, and their production limitations, were discussed. S. Malekmohammmadi et al. reviewed stimuli-responsive hydrogels based on their external triggers, and their applications in biomedical fields [45]. 3D printing techniques used for manufacturing hydrogels were briefly summarized. A. Samandri et al. reported a systematic review of natural bio-inks developed by 3D printing for skin regeneration and wound healing [46]. The authors found that collagen and gelatin hydrogels were the most commonly used bio-inks for wound healing applications. Recently, M. Nadhif et al. briefly reviewed 3D printed wound dressings. The study found that alginate, among the hydrogels, was the most common hydrogel used for wound dressings, and pneumatic FDM, among the 3D printing mechanisms, was the most popular mechanism [47]. The authors stated that 3D-printed hydrogel wound dressings have never been reviewed. This review focuses particularly on 3D printing of smart hydrogel wound dressings which can perform multiple functions, such as delivering drugs and monitoring wound site conditions, in addition to their primary functions. Unfortunately, these have been rarely mentioned in previous literature, to the best of the authors’ knowledge. The shortcomings of traditional wound dressings are summarized. In addition, the advantages of modern hydrogel wound dressings are highlighted. Commercially available 3D printing techniques used for developing hydrogel wound dressings are discussed in detail and the challenges involved in manufacturing are considered. Recent advances in smart hydrogel wound dressings are covered in detail. Additionally, hydrogel wound dressings which are integrated with sensors for monitoring wound site conditions are discussed. Furthermore, the future potential of smart wound dressings developed using 3D printing is discussed.

## 2. Skin Structure

The skin defends against external, chemical, and biological factors [48]. In addition, it prevents water loss from the body (dehydration) and maintains temperature regulation. Healthy skin has a weakly acidic pH which assists in combating harmful microbes and damaging free radicals that might increase the aging process. Several factors may alter the skin’s pH level, such as skin exposure to environmental factors (e.g., air pollution and humidity levels), some detergent antibacterial soaps and gels, sweat, and longtime exposure to the sun. When the skin is subjected to an injury, the pH level changes to alkaline due to release of exudate from the tissues. The human skin consists of three layers: the epidermis, the dermis, and the hypodermis which are discussed in more detail in the next section (Figure 1a). 

### 2.1. Epidermis

The epidermis is the visible outer layer of the skin that provides protection for the body. The skin has a barrier function, containing keratinocytes that form the epithelium, including basal keratinocytes in the innermost layer, and keratinized tissue [49]. The basal keratinocytes undergo regular proliferation to reconstruct the whole epidermis to ensure the epidermis renews. There is a basement membrane underneath the epidermis which separates the epidermis from the dermis.

### 2.2. Dermis 

The dermis layer is located beneath the epidermis and has a thickness of 1–4 mm. Fibroblasts are the primary cells in the dermis providing the dermis with mechanical strength and elasticity [49]. The dermis layer includes fibroblasts, neutrophils, mast cells, and dermal dendritic cells. The dermis contains various structures, such as sweat glands, hair follicles, sebaceous glands, and nerve endings [50]. In addition, it contains substantial networks, such as nerve, blood, and lymphatic vessels [49]. The dermis is made of two layers: the papillary and the reticular dermis [49]. The papillary dermis is the top layer and contains the connective tissues and the blood vessels that deliver nutrients to the epidermis [48,49]. The reticular layer is located below the papillary dermis and consists of thick collagen and elastic fibers which give the skin strength and elasticity [49]. 

### 2.3. Hypodermis

The hypodermis is the subcutaneous layer located below the dermis and consists mainly of fat. The hypodermis prevents heat loss from the body by providing thermal insulation between the skin and skeletal structures [50]. The thickness of the hypodermis varies in different regions of the body and between different individuals. Initially, the hypodermis was viewed as a tissue used for fat storage, but, later, it has been found that it serves many important functions. These functions include hormone production, body temperature regulation, and protection.

## 3. Skin Wounds

The Wound Healing Society divide chronic wounds into four categories: pressure ulcers, diabetic ulcers, venous ulcers, and arterial insufficient ulcers. These categories share common characteristics, such as persistent reactive oxygen species (ROS), senescent fibroblasts, prolonged infection, and dysfunctional and insufficient stem cells [51].

### 3.1. Common Skin Wounds and Clinical Treatment 

A skin wound refers to damage at the surface of the skin. The typical clinical medication is suturing (stitches) if there is an open split or gaping has occurred. Wound healing is a complex and dynamic process which involves the repair of cellular structures and tissue layers. The healing rate and the process depend on whether the wound is acute or chronic. Table 1 and Table 2 summarizes the common wound types, their causes, clinical treatments, and the characteristics of each treatment.

### 3.2. Acute and Chronic Skin Wounds

Wounds can be caused by physical, chemical, and thermal damage. There are two types of skin wounds based on their healing period and healing ability: acute and chronic wounds.

#### 3.2.1. Acute Wounds

Acute wounds may last for 8 to 12 weeks accompanied by a substantial exudate, heavy infections, pain, and tissue necrosis [56,57,58,59,60,61,62]. Acute wounds are mainly caused by mechanical injuries, such as frictional contact between the skin and hard surfaces, such as knives, penetration of gunshots, and surgical incisions. They may happen due to chemical and burn injuries, radiation, corrosive chemicals, electricity, and thermal injuries [62]. Acute wounds are characterized by common bacteria, low inflammatory cytokines, and high mitogenic activity [63].

#### 3.2.2. Chronic Wounds

Chronic wounds take more than 12 weeks to recover [64]. These wounds are mostly caused by repeated insults to skin tissues or exposure to physiological conditions, such as diabetes, impaired angiogenesis or innervation, or cellular migration [64]. Some related factors are malignancies, infections, poor primary treatment, and other patient-related factors [65]. Examples of the most common chronic wounds include, diabetic foot ulcers, pressure ulcers, and venous leg ulcers [66]. Bacterial infections, impaired immune function, and serious health conditions, increase the risk for developing chronic wounds. Additionally, diagnosis with diabetes or cancer may increase the risk for developing chronic wounds. Wound dressings can accelerate the wound healing rate for both acute and chronic wounds. In some instances, wound dressings may deliver and control release of drugs or growth factors [67]. Chronic wounds are characterized by contamination with high levels of bacteria, high inflammatory cytokines, and a degrading nonfunctional matrix [68]. The methods used to identify the wound type are summarized in Table 3.

**Table 3:** Some methodologies for identifying a chronic or acute wound.

Gauge used	Measurement	Indication	Ref.
iDr or mobile app.	3D imaging of the wound (Figure 2a)	By applying the optical imaging principle and surface feet per minutes (SFM), using a smartphone video, iDr can accurately and non-invasively reconstruct a 3D wound model and measure the wound’s area and volume in 3D digital space. Using recorded history data on volume and area, iDr can help clinicians analyze wound healing effectiveness during treatment.	[69]
Matrix metalloproteinase (MMP)	Collecting wound fluids (22 samples) and chronic wounds (25 samples) of various etiologies, including mixed vessel disease ulcers, decubitus and diabetic foot ulcers (Figure 2b).	Chronic wounds (median 22.8 μg MMP Eq/mL) compared to acute wounds (median 0.76 μg MMP Eq/mL) (*p* < 0.001).	[70]

**Figure 2:**
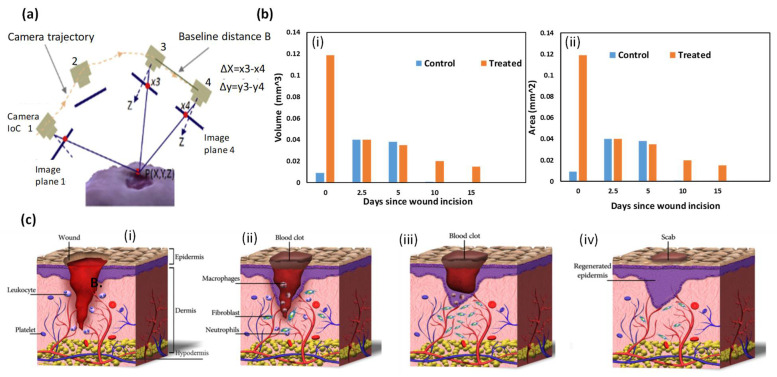
(**a**) iDr or mobile app., (**b**) matrix metalloproteinase (MMP), (**c**) skin wound healing process: (**i**) hemostasis (blood clotting), (**ii**) inflammation, (**iii**) tissue growth (proliferation), and (**iv**) tissue remodeling (maturation) [68,69,70].

### 3.3. Skin Wound Healing Process 

The short wound healing process follows specific biological steps. When the injury begins to heal, the blood platelets are activated to form a blood clot and play a role in leukocyte recruitment [58]. Then, neutrophils and macrophages remove dead or impaired cells, external bacteria, and other debris located in the wound site. Next, fibroblasts migrate, proliferate, and activate the angiogenesis process. After that, granulation tissue is formed, extracellular matrix proteins are deposited to reconstitute the dermal tissue, and the epidermis is regenerated [71]. Finally, the formed capillaries and fibroblasts are aborted. The four phases occurring during the wound healing process are summarized in the next sections (Figure 2c).

#### 3.3.1. Hemostasis (Blood Clotting)

Hemostasis is a body response in which the platelets and the inflammatory cells aggregate at the wound site. It is accompanied by the release of clotting factors [56,58]. A few minutes after injury, blood platelets stick together, adopt an amorphous shape, and aggregate at the wound site (Figure 2c(i)). Platelets play a crucial role in leukocyte recruitment, and in the initiation and progression of inflammation [71]. 

#### 3.3.2. Inflammation

Inflammation occurs after or during the hemostasis stage, once the fiber clotting system is formed. Blood monocytes and lymphocytes differentiate the tissue macrophages at the wound site, releasing growth factors [72]. In this phase, immune cells, particularly neutrophils and macrophages are released into the wound site (Figure 2c(ii)) [50]. The inflammatory cells and platelets release different peptides and growth factors, which initiate formation of fibroblasts in the wound site and activate angiogenesis [73]. 

#### 3.3.3. Tissue Growth (Proliferation)

The tissue growth phase begins after 2 to 3 days of the injury and lasts until wound closure. In this stage, fibroblasts differentiate into myofibroblasts that seal the injured area by pulling the wound edges together [74]. During the releasing phase, the fibroblasts are further stimulated to proliferate in the wound area. This phase reconstructs the dermal tissue components by forming granulation tissue and deposition of extracellular matrix proteins, mainly collagen [75]. Enhanced angiogenesis induces ingrowth of a new network of blood vessels (Figure 2c(iii)). Epithelial cells migrate from the wound edges to cover the defect, a process known as ‘epithelialization’. During the proliferative phase, the granulated tissues are constructed from epithelial cells and fibroblasts, and the keratinocytes are altered to extracellular matrix (ECM) [76].

#### 3.3.4. Tissue Remodeling (Maturation)

In the maturation phase, excess collagen fibers are degraded in the dermis and wound contraction begins to peak. The healed wound reaches 80% of the original ultimate mechanical strength [77]. The human skin consists of fibroblasts, keratinocyte cells, and collagen matrix that together form dermis and epidermis-like structures (Figure 2c(iv)). Previously, to achieve compatibility, researchers produced embedded fibroblasts and keratinocytes into the collagen matrix to form dermis and epidermis-like structures. For instance, researchers reported introducing a composite made of embedded fibroblasts and keratinocytes into the collagen matrix, using 3D printing. More details are given of the wound healing processes in [77].

## 4. Smart Hydrogel Wound Dressings

Hydrogels are three-dimensional crosslinked hydrophilic polymers which are water insoluble. They imbibe and retain a large amount of water while maintaining their defined structures [18]. Hydrogels contain at least 10% water of their total volume/weight [78]. Hydrogels are very flexible in a similar way to human tissues because of the considerable water content. The hydrophilicity of a hydrogel network results from the hydrophilic functional groups distributed in the structure, such as hydroxyl, carboxylic, amine, sulphonyl hydroxide, and amide groups [78]. Hydrogels possess distinctive properties, including tunable mechanical strength, sensitivity to external stimuli, and high oxygen and water permeability [79].

Hydrogels can be classified into two categories: natural and synthetic hydrogels. Natural hydrogels are suitable for biomedical applications as they are biocompatible, biodegradable, and biologically recognizable moieties [80]. They involve collagen, fibrin, hyaluronic acid, and derivatives of natural materials, such as chitosan, alginate, and skill fibers [80]. They are physiological hydrogels as they mimic the extracellular matrix (ECM); however, they may trigger immune/inflammatory responses if introduced to the human body [81]. Natural hydrogels show a significant improvement in healing rate, rate of granulation, and repair of impaired blood vessels [81]. However, the main drawbacks of natural hydrogels are the challenges related to reproducibility of their final microstructures [82]. Examples of typical natural hydrogels incorporating chitosan or alginate are chitosan/polyethylene glycol (PEG) hydrogels, chitosan/PEG/poly(vinyl pyrrolidone) (PVP) coated cotton fibers, chitosan/poly(vinyl alcohol) (PVA) sponges and hydrogels, chitosan/PVA/poly(ethylene oxide) (PEO) hydrogels, carboxymethyl chitosan/gelatin hydrogels, and chitosan/lactic acid aerogels [83,84,85,86,87,88,89]. 

In contrast, synthetic hydrogels, such as poly(ethylene glycol) diacrylate, poly(acrylamide), and polyvinyl alcohol, are more reproducible [86]. However, their final structures depend on the polymerization conditions. So, rigorous control of the preparation protocols, including temperature and environment control, are necessary [87]. Generally, synthetic hydrogels offer more flexibility for tuning their chemical composition and mechanical properties. For example, modifying precursor concentration, molecular weight, and percentage of the used crosslinker, may optimize mechanical properties and other favorable properties. Synthetic hydrogels can also be selected or tuned to be hydrolysable or biodegradable over variable periods. However, synthetic hydrogels do not have any inherent bioactivity. 

Smart hydrogels can respond to various stimuli, such as temperature, pH, electric and magnetic fields, light intensity, and biological molecules [88]. The stimuli generate macroscopic responses in the material, such as swelling or collapse [89]. Smart hydrogels comprise intelligent cross-linked networks that undergo chain reorganization from collapsed to expanded. They also retain smart surfaces that transform their hydrophilicity as a function of the stimulus-responsive interface. In some circumstances, linear and solubilized smart macromolecules pass from monophasic to biphasic, giving rise to reversible sol-gel hydrogels [90]. For example, temperature-sensitive hydrogels of low critical solution temperature (LCST) or upper critical solution temperature (UCST) depend on temperature for their transition behavior from monophasic to biphasic or vice versa [91]. pH-sensitive hydrogels such as poly-acidic moieties (poly(acrylic acids), and poly(methacrylic) acids), or polybasic moieties (poly(N-dimethylamino ethyl methacrylate), poly(N-diethylaminoethyl methacrylate), and poly(ethyl pyrrolidine methacrylate)), protonate or deprotonate as a response to the surrounding pH [92]. Photo-sensitive hydrogels experience a reversible or irreversible transformation in conformation, polarity, amphiphilicity, charge, optical chirality, and conjugation in response to a light stimulus [92]. Reversible chromophores or molecular switches undergo a reversible isomerization upon light irradiation [93], while irreversible chromophores are cleaved from the polymer chain upon light exposure.

In addition to physical stimuli, biological materials which contain receptors for biomolecules undergo modification in material properties when stimulated. An example of these smart biological hydrogels are enzyme-responsive hydrogels that respond to selective enzyme catalysis [93]. These materials represent a significant advancement in integrating artificial materials with biological entities. Enzyme responsive hydrogels can also display reversible and dynamic responses to a stimulus. 

In 1970, the first moistened wound dressing was introduced, which overcame some problems of traditional dressings [94]. Hydrogel-based wound dressings have unique characteristics as they mimic the native skin microenvironment. Researchers found that wounds covered with hydrogel bandages heal faster. Moreover, hydrogel bandages can be applied to some body parts which traditional wound dressings cannot reach [94]. Introducing hydrogel bandages assists in keeping the wound moistened, enhances oxygen permeability, and absorbs wound exudate, protecting the wound from external pathogens and contaminations that occur during the wound healing process. In addition, hydrogel dressings can be loaded with biomarker indicators which are sensitive to external stimuli and function smartly [95,96]. Hydrogel wound dressings are hydrophilic porous structures capable of absorbing large amounts of water as they contain hydrophilic groups, such as -NH_3_, -COOH, -OH, -CONH_2_, -CONH, and -SO_3_H, that can form polar to polar adhesion with water chains. In addition, they can deliver water to the wound site, with their moisture content, non-adhesive nature, and malleability similar to that of living tissues [97]. In some instances, a highly absorptive wound dressing is very desirable, such as in the case of venous leg ulcers which produce an extensive amount of exudate [98]. Furthermore, hydrogel bandages modulate transmission of gases and ions to the wound site [97]. Hydrogel wound dressings assist in stopping bleeding, relieve pain, and provide mechanical protection. They absorb excess exudate, keep the wound moist, and dissolve necrotic tissue and fibrin. Furthermore, they are easy to attach to healthy skin, and do not adhere to neoformative granulation growing tissues, avoiding secondary injuries during dressing replacement [97]. They can also protect new growing tissues from damage during the taking-off and covering of the wound. There is also a great need to be able to load active drugs and antibiotics into wound dressings to assist in healing chronic or complex wounds. The specifications for an ideal wound dressing include plentiful water vapor, oxygen permeability, biocompatibility, nontoxicity, infection protection, and acceleration of the formation of granulation tissue and epithelialization rate [99,100,101,102]. Hydrogel dressings can cool down the wound and reduce pain, which makes them very beneficial for burns or painful wounds [103]. All these properties of hydrogel dressings make them the best option for wound dressings. However, available hydrogels may exhibit very low tensile stresses that are not compatible with load-bearing applications.

Wound dressings can be made of natural and synthetic hydrogels (more information can be found in the cited references [80,81,82]). Roel et al. reviewed the types of hydrogel skin wound dressings and the materials they are produced from, including natural and synthetic. Some examples are given in Table 4 [104]. 

### Effect of the Hydrogel Crosslinking Process on Mechanical Strength and Water Absorption

Hydrogel-based wound dressings generally contain 5 to 10% of crosslinking polymers [88]. Crosslinking is the stabilization process in which a polymer makes multidimensional extension chains. The crosslink is the bond that links one polymer to another. Crosslinking can be measured by determining the swelling ratio or the water absorption rate. The more crosslinked the hydrogel, the less swelling is attained, however, the higher the strength. Increasing the crosslinking ratio potentially increases the strength; however, if crosslinking is excessive or in a high ratio, the material becomes rigid or glassy. The cross-linker can be a physical crosslinker formed by weak interactions that constitute a bridge between the bonds of the polymers, or a chemical crosslinker, formed by covalent bonds which are tough to degrade [117]. Crosslinking works by crossing the polymer chain as some polymers may lose their ability to move as an individual chain. The different types of crosslinking, illustrated with some examples, are summarized in Table 5.

**Table 5:** Types of crosslinking mechanism.

Type of Crosslinking	Monomers	Common Crosslinkers	Ref.
Homopolymer (single network)	Poly(2-hydroxyethyl methacrylate) (Figure 3)	Polyethylene glycol dimethacrylate	[90,93]
		Triethylene glycol dimethacrylate (TEGDMA)	
Co-polymer (double or more)	Polyethylene glycol (PEG)/		
	methacrylic acid (MAA) (Figure 3)	Tetra(ethylene glycol) dimethacrylate	[90,92,122]
	Carboxymethyl acid cellulose (CMC)/		
	Poly(vinyl pyrrolid) (PVP)		
Semi-interpenetrating network (semi-IPN)	Acrylamide/acrylic acid copolymer/ Linear cationic polyallylammonium chloride	N,N’-methylene bisacrylamide	[93]
Interpenetrating network (IPN)	Poly(N-isopropyl acrylamide)/ Chitosan	N,N’-methylene bisacrylamide	[123]

**Figure 3:**
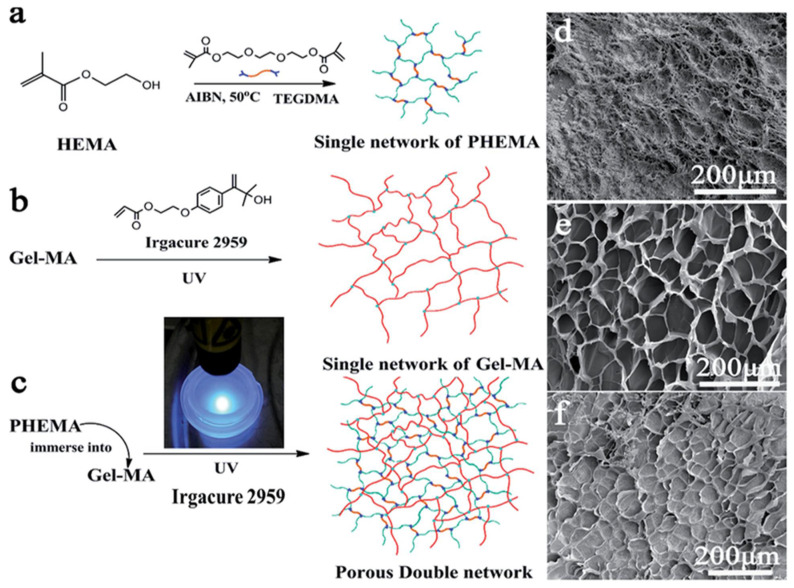
(**a**) Single network of pHEMA, (**b**) single network of Gel-MAA, (**c**) porous double network, (**d**) SEM image polyHEMA, (**e**) SEM of polyMAA, (**f**) SEM of the co-polymerized HEMA/MAA.

Most of the hydrogels mentioned in Table 5 are involved in drug delivery and tissue regeneration applications. Among the different crosslinking types, the semi-IPN type effectively responds to pH or temperature due to their restricting interpenetrating elastic network [92]. In addition, they possess characteristics such as modified pore size and slow drug release. Double network (DN) hydrogels have a lower water absorption rate, and higher mechanical strength and toughness. The first network, the minor component, comprises abundantly cross-linked polyelectrolytes (rigid skeleton) and the second network, the primary component, comprises poorly cross-linked neutral polymers (ductile substance). Haque et al. found that the specific combinations of two networks with contrasting structures were the main reason for the high mechanical performance of biomaterials [124]. The first network is sacrificial bonds that effectively dissipate the stress and the second ductile polymer chain can extend extensively to sustain large deformation [124]. The primary and secondary networks in the double network are responsible for the mechanical stability. At high strain, the primary network is irreversibly destroyed preventing the secondary chain from failing. Characteristics of double network hydrogels are summarized in Table 6.

## 5. Additive Manufacturing/3D Printing

**Figure 4:**
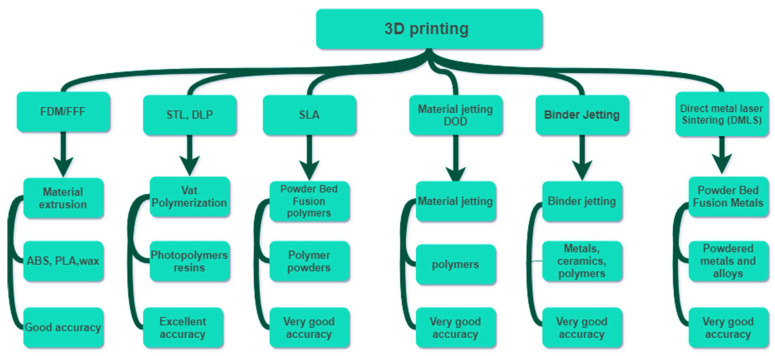
Classification of 3D printing based on printing techniques, material usage, and accuracy.

3D printing technology has been popularized over recent years [126,127]. Each 3D printing technique, i.e., fused deposition modeling (FDM), stereolithography (SLA), polyjet process, selective laser sintering (SLS), 3D inkjet, and digital light processing (DLP), has different characteristics (Figure 4). The characteristics involve repeatability, resolution, and accuracy, printing time, and the ability to process different raw materials. Table 7 summarizes the main characteristics for each 3D printing technique [128]. There is a compromise among 3D printing techniques in terms of materials used, resolution, repeatability, accuracy, and hence in their applications. Compared to conventional techniques, 3D printing requires fewer steps and less manual labor to produce intricate prototypes [128]. Moreover, some of the essential advantages of 3D printing include its simple fabrication process, quick production, low waste generation, and risk mitigation [129]. The advent of 3D printing in wound dressings has shown promising outcomes through overcoming several challenges. The promising features of 3D printing in wound dressing applications result from the capability of 3D printing to control and design sub-micro components of the printed bandages. All 3D printing techniques have been shown to be helpful over a specific range; however, inkjet printing and DLP were found to be capable of providing prototypes with the highest repeatability [128]. The most commonly used 3D printing techniques for skin wound dressings, are digital light processing (DLP) and stereolithography (STL) due to their ability to process biocompatible polymer materials, such as hydrogels, that can mimic the ECM of the skin structure. DLP and STL have additional advantages, such as high accuracy, smooth surface finish, high resolution, and high repeatability (Table 7) [103]. However, DLP and STL suffer from challenges, such as being unable to print large structures with good mechanical properties, and boxy surface finishes [128]. 

### 5.1. Recent Bio-Printing Technologies Outcomes and Limitations

Burn treatment, especially in the case of extensive burn injuries, involves surgical excision of the injured skin and reconstruction of the burn injury with skin substitutes. To accomplish the substitution of the skin, bioprinting methodology is applied. Skin bioprinting is the most recent and advanced wound treatment in the clinical field. Bioprinting for reconstructing burn injuries, involves layer-by-layer deposition of cells and scaffolding materials over the injury area. Bioprinting is a reproducible fabrication technique that enables accurate placement of cell types. Traditional dressings work to stop bleeding and to seal the wound from external pathogens but bioprinting-based wound dressings aim to achieve wound closure, and to improve scar quality and functional outcomes [147]. Bioprinted wound dressings are usually produced for small to moderate-sized burn scars and consider Langer’s skin tension lines to achieve optimal esthetic outcomes [148]. The bioprinting procedure for manufacturing a wound dressing is the same as for traditional 3D printing. The wound site is scanned by computed topography (CT) or magnetic resonance imaging (MRI) and the image is converted to a CAD model. This is followed by selection of the appropriate biomaterial and cells. Finally, the 3D printed cells are applied to the wound directly. In bioprinting, an adequate cell donor is needed. Inkjet bioprinting, micro-extrusion bioprinting, and laser-assisted bioprinting are standard 3D printing techniques for developing wound dressings [149]. The desired shape could be printed in a liquid container- or a solid container-3D printer. Stabilizing the final shape using UV light, and other chemical and physical processes are quite often needed after 3D printing. A post-printing process, such as tissue maturation in a bioreactor, animal impanation, or in vitro testing, is necessary.

There are some technical limitations with bioprinting at the pre-printing stage, during bio-printing, and during maturation stages [148]. Some of the main challenges with bioprinting are the need for multiple cells, the biomechanical properties for the clinical translation, and the high printing resolution required to replicate inner microarchitectures [150]. Optimizing printing parameters, such as the dispensing pressure, printing time, nozzle diameter, extrusion speed, laser energy, substrate film thickness, viscosity, droplet size, and cell differentiation, are other challenges. Building a functional vasculature and producing a bio-ink which conform to the native skin of different cell types, requires different nutritional and metabolic support. Additionally, there is a knowledge gap with respect to post-printing of cellular dynamics, fusion, deformation, and the stiffness of bio-printed parts [148]. 

Skin closure could also be performed using skin stretching devices which can cover the excised burn wound with autologous skin harvested from an uninjured donor site. Despite promising clinical results, there are still many challenges regarding skin substitutes. For example, most skin substitutes consist of allogeneic skin which can be highly immunogenic and contain cellular remnants that may cause body rejection for the skin substitute [151]. In addition, methods to sterilize skin substitutes may be insufficient to eliminate transmission of unknown or prion disease from animal material [152]. Furthermore, human-derived skin is limited by its supply and the structure of human skin is more complex than biosynthetic substitutes. Finally, although most skin substitutes perform relatively well in the clinic, these substitutes do not include hair follicles and pigments, which are critical for the skin to function normally [149]. Grafting can be considered the gold-standard type treatment for some cases; however, in some instances, patients may not have enough skin available for grafting due to extensive burns. In addition, in some cases, immune system rejection or virus transmission may occur [153].

### 5.2. D Printed Hydrogel Patches by DLP/SLA

**Figure 5:**
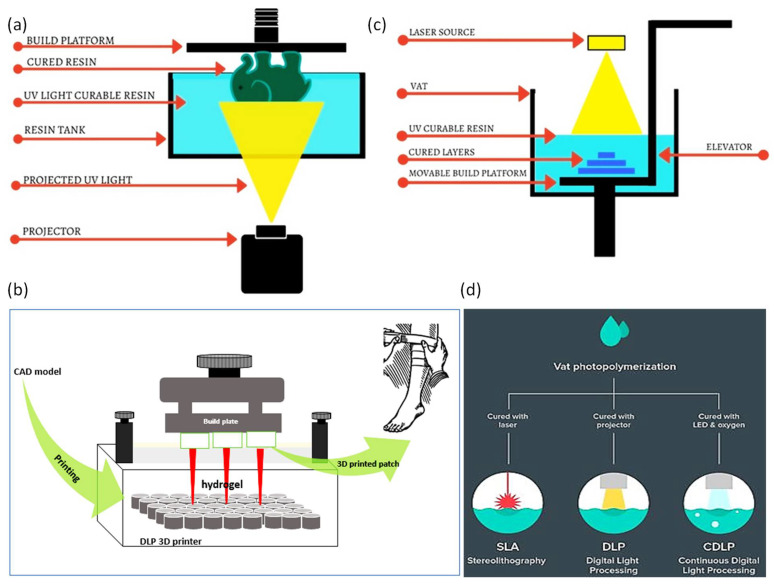
(**a**) DLP printer, (**b**) DLP printing general strategy, (**c**) SLA, and (**d**) photo-polymerization of DLP and SLA technologies.

Stereolithography (SLA) and digital light projector (DLP) printers are based on containers that hold photo-curable liquid resins, laser sources or UV-light to induce polymerization, with dynamic systems allowing 3-dimensional movements (Figure 5). The fundamental differences between SLA and DLP printers are that DLP printers use a UV-light from a projector energy source, while SLA printers use a UV-laser beam source [154]. The DLP cures the resin layer at one point in time, but the SLA uses a point-by-point curing technique. As a result, DLP printing saves time and cost compared to SLA printing [154]. Both SLA and DLP have advantages in the wound dressing manufacturing field. 3D printed wound dressings made by SLA/DLP techniques can be loaded with antibacterial nanoparticles, antibiotics, and some other biological substances. Each level of the printing is fully controlled in situ to produce the desired part. The DLP process prints successive layers by lifting the platform equal to the thickness of the part. The main reason for curing a whole layer at once is the dynamic mask that combines liquid crystal display (LCD), spatial light modulator (SLM), digital micro-mirror device (DMD), etc. The dynamic mask carries the design pattern in which the light passes and transmits the pattern to the receiving substrate [155]. Though additive manufacturing has shown promising results in manufacturing the bandages and has reduced the difficulty of traditional wound dressings, some issues still need to be resolved.

**Figure 6:**
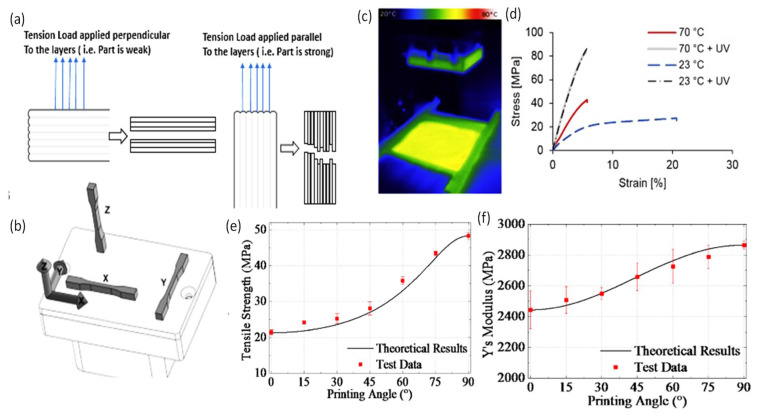
(**a**) Tension load applied perpendicular to the layers, (**b**) tension load applied parallel to the layers, (**c**) the printing orientation, (**d**) the correlation of the temperature and the stress [158], and (**e**,**f**) the correlation between the tensile strength, Young’s modulus, and the printing orientation [160].

In both SLA and DLP techniques, after completing the curing of the first layer, the platform goes down, so that a new layer of the resin covers the previous layer. In DLP printers, a digital micro-mirror device (DMD) includes an array of millions of independently rotatable micro-mirrors that can generate an image of a cross-sectional layer on the resin vat and all the targeted points are cured at once, which makes the DLP faster than the SLA technique [126,156]. However, in the SLA, the laser spot provides a mini-feature size. Objects printed via SLA have a better spatial resolution than DLP, mainly due to the SLA laser’s small spot size. Further details on the difference between STL and DLP printers, are discussed in [157]. 3D printers work by creating successive layers using a photo- or thermal-polymerization process. As a result, proper adhesion is necessary between the layers, and in some instances, this generates a problem if the printed part contain voids or is overhung. The low quality of layer adhesion is the main reason behind the poor mechanical properties of printed wound dressings. Additionally, light is attenuated as it propagates to the resin, so the light intensity decreases with the resin volume and insufficient polymerization may be induced [158]. In the DLP printing technique, the resin monomer should be filled above 40% of the resin tray, and its volume requires to be checked by a built-in sensor before the printing process is initiated. After printing, the supporting material of the printed part should be appropriately removed without destroying the surface features. Then, the samples need to be washed with isopropyl alcohol (IPA) to remove leftover unreacted resins, followed by a UV bath to solidify the part if needed. Alketbi et al. reported 3D printed polyethylene glycol diacrylate (PEGDA) hydrogel that showed high elasticity and irreversible densification. Pore formation in the hydrogel was highly dependent on the exposure time, light intensity, and the associated degree of crosslinking [159]. Sherman et al. studied the effect of resin viscosity, orientation, and spacing of the pores in DLP printing [157]. At higher viscosity, Newtonian behavior appeared, and the porosity of the printed part was slightly reduced from theoretical values by 7% [158]. Steyrer et al. showed that higher printing temperatures produce higher double-bond conversion and tensile strength (Figure 6) [158]. However, printing temperature did not affect the properties after post-curing in the XYZ orientation [158]. High printing temperatures decreased the viscosity of the resin reducing the printing time and provided better mechanical properties while post-cured properties were found to be unaffected [158]. Many factors need to be considered when selecting DLP and SLA printers, which include the part size, printing speed, scanning speed, layer thickness, laser type, optical system, operating software, CAD interface, machine size, ambient temperature, relative humidity, and the power.

## 6. Recent Developments in the 3D Printing of Hydrogel Wound Dressings 

The most common 3D printing technologies employed for printing hydrogels, are direct ink writing (DIW), DLP, and SLA [161]. UV-curable polymers are widely used, including elastomers, rigid polymers, acrylonitrile butadiene styrene (ABS), and polylactide acid (PLA) polymers; however, they are water-insoluble. On the other hand, PEGDA and acrylamide/PEGDA hydrogels are highly stretchable, and have high water retention capability [161]. Cascone et al. discussed conventional or acellular hydrogel-based commercial wound dressings for biomedical applications [129]. Some of the standard commercial hydrogel-based wound dressings are listed in Table 8.

Yongji et al. produced a paper on a photo-curable hybrid chitosan/acrylamide bio-ink for DLP-based bio-printing [156]. The hydrogel characteristics were analyzed by carrying out mechanical testing, scanning electron microscopy (SEM) analysis, and swelling tests. In the mechanical test results, the several ratios of the co-polymerizing covalent crosslinking between the chitosan modified with methacryloyl groups (CHIMA) and acrylamide (AM), significantly affected mechanical strength differences. Proportions of 20 wt% of pAM and 1% of CHIMA showed the highest tensile and compression strength [156]. The results indicated that the higher the degree of hydrogel crosslinking, the higher the mechanical strength. Unlike other published outcomes, a higher swelling ratio for the highly crosslinked polymer was observed in this study. However, the involved paper limited any actual application due to a decrease in the adhesion and the compatibility with the dynamic skin motion. Muwaffak et al. developed wound dressings using stereolithography [163]. The fabricated patches were made of polyethylene glycol diacrylate (PEGDA) laden with metals, including silver, copper, and zinc polycaprolactone. Diphenyl(2,4,6-trimethylbenzoyl)phosphine oxide (TPO) was used as the photo-initiator. The wound dressings were capable of preventing wound infections due to the anti-mycobacterial properties of the incorporated metals that enhanced wound healing. The dressings were shown to have fast- and slow-release properties. Using this technique, six drugs, including paracetamol, caffeine, naproxen, chloramphenicol, prednisolone, and aspirin, were incorporated into the 3D printed bandages with different geometries and material compositions [163]. However, the adhesion of the printed bandages and their applicability to some unreached parts of the body were still limited. Cereceres et al. produced 3D printed hydrogel wound dressings loaded with a novel antimicrobial (gallium maltolate) to prevent chronic wound infection [164]. The gallium maltolate rapidly leaked out when the dressings contacted water. The shortcoming of the produced wound dressing was that it could not be applied on wet wounds. Nizioł et al. produced antimicrobial wound dressings by 3D printing based on thermo-responsive hydrogel [165]. Nanofibers were prepared via electrospinning with controlled relative humidity and surrounding temperatures. Then, a temperature-controlled pneumatic-based extrusion printer was used to print the wound dressings. Nanofibers with a maximum breaking strength of 0.06 MPa were obtained at the maximum crosslinking time (8 h). The bandage’s stretch ability provided a robust solution for the joint section of the skin. The researchers correlated the swelling ratio, temperature, and time of the developed bandages to optimize the specifications. The wound dressings were capable of sensing the temperature as the printed ink contained poly (N-isopropyl acrylamide) (PNIPAAm). Although the dressings had good stretchability, they suffered from low mechanical strength due to the poor layer-on-layer adhesion. Milojevic et al. developed a hybrid, extrusion-based 3D printed hydrogel for wound dressing [166]. Polycaprolactone and alginate/carboxymethylcellulose gels were printed layer-by-layer. The analysis of the printed parts showed fine-tuned wettability (50–75%), an enhanced swelling ratio and mechanical strength (11 MPa). Moreover, the dressings were found to have a reasonable degradability rate. However, the adhesion technique and its applicability to bending joints, such as knees, are still a challenge. Liu et al. developed tough hydrogel patches by incorporating CaCl_2_ [167]. The results showed an increase in the concentration of Ca^2+^ was linked with a decrease in the water content leading to an enhanced Young’s modulus. In addition, a modified pH phenol red dye was entrapped in the hydrogel patches to show change in pH by indicating the color. Navarro et al. reported that the printing orientation has a statistically significant effect on the compressive modulus independent of the printer type [168]. The analysis showed that the horizontal structure exhibited a higher Young’s modulus than for the vertical structure [168]. It is also expected that structures with high porosity possess low compressive moduli. Additionally, as the tilt angle increased, the print time increased, subsequently, the building layers increased [82]. It is well-known that the AM process demonstrates microstructural anisotropy due to the layer-by-layer nature of the production. Mueller et al. conducted intensive experiments to correlate the printing orientation with the ultimate tensile strength and elastic modulus [169]. It was found that the horizontal orientation had the highest ultimate tensile strength and elastic modulus. During sample printing, the laser light used for polymerizing the new layer passes through previously printed layers, so the light is attenuated with the penetration depth. If the thickness of the printed layers is thick enough, the laser might not be sufficient to induce photo-polymerization [170].

Furthermore, 3D printing of hydrogels exhibits some compelling features. H. Baniasadi et al. reported 3D printed hydrogels that showed a very high porosity with open and interconnected pores which allowed for a high-water uptake capacity (up to 1600%) [171]. Composite 3D-printed materials open options for skin wound dressing applications due to their mechanical strength that is comparable to that of soft tissues. Drug-loaded dressings demonstrated controlled and efficient delivery of the antioxidant, ascorbic acid, in phosphate-buffered saline (PBS) at 23 °C, where 80% of the loaded drug was released within 8 h [171]. The experimental findings indicated a correlation among shrinkage, porosity, pore size, and the swelling ratio of the dressings. The shrinkage of the dressing was reversibly proportional with the pore size. However, the mechanical strength of the developed dressing was not as high as the commercial non-hydrogel polymers but good water absorption was observed for the reported dressing. Y. Ren et al. conducted in vivo tests on rat models using tannic acid/keratin hydrogel wound dressings crosslinked by graphene oxide quantum dots and citric acid [172]. The results revealed complete skin wound healing in 16 days [172]. The printed dressings showed promising results; however, more investigation is still needed prior to clinical testing [172]. Several research groups have shown that use of micro gels has similar effects to hydrogel dressings. S. Hou et al. prepared a gelatin microgel and injected it directly into a cut section of a porcine cornea along with the crosslinker microbial transglutaminase [173]. The microgels turned to microporous hydrogel upon crosslinking, allowing cell migration and controlled release of growth factors [173]. Likewise, X.Zhang et al. used methacrylate hyaluronic acid and modified alginate microgels to form macroporous gels under alkaline conditions. These gels were shown to be effective for cell migration in the growth of blood vessels tested on rat models. Y.Ashoori et al. treated chitosan nanogels with probiotics which promoted wound healing during in vivo tests on rat models [174].

Y. Hu et al. used an extrusion-based cryogenic 3D printing technology to construct decellularized small intestinal submucosa (SIS) integrated with a mesoporous bioactive glass (MBG) and exosomes to produce a 3D scaffold dressing (SIS/MBG@Exos) that was capable of sustained release of bioactive exosomes [175]. The fabricated SIS/MBG@Exos hydrogel scaffolds were shown to possess a 3D structure with appropriate porosity, biocompatibility, and hemostasis capability. The results of testing the scaffolds on diabetic wounds, confirmed that hydrogel scaffolds can accelerate diabetic wound healing by increasing blood flow and promoting the angiogenesis process of the diabetic wound [175]. H. Chen et al. reported polyethylene glycol (PEG) wound dressings loaded with silver nitrate (AgNO_3_) and the angiogenic drug, desferrioxamine (DFO) [176]. The dressings were tested on rat models and were shown to resist myriad external forces, such as squeeze and twist, and stayed in their initial shapes. Kumar et al. developed chitin hydrogel nano ZnO composite bandages [177]. The homogenized mixture of chitin hydrogel and nano ZnO was freeze-dried to obtain microporous composite bandages. The nanocomposite bandages showed enhanced swelling, blood clotting, and antibacterial activity [177,178]. J. Leppiniemi et al. demonstrated that an alginate/nanocellulose hydrogel wound dressing developed using 3D printing had good mechanical properties and tissue compatibility [179]. Some examples of common nanocomposite hydrogel wound dressings are listed in Table 9.

## 7. D Printed Wound Dressing Integrated with Sensors

Along the healing process of the wound, non-contact, low-cost, effective and remote monitoring sensors are needed. Biosensors are devices that can deliver analytical/biochemical information from the system [188]. This may significantly reduce the need for the assistance of clinicians in diagnostics and treatment. Hydrogels are smart materials that can respond to either physical stimuli, such as temperature, electric and magnetic fields, light intensity, and pressure, or chemical stimuli, such as pH, ions, and specific chemical compositions. A fascinating feature of hydrogels is their ability to return to their original size once triggers are removed.

Biomarkers or biological biomarkers refer to what is happening to an organism at an instant moment [188,189]. Biomarkers show symptoms resulting from a disease or effects of a treatment. The biomarker-driven approach shortens clinical trial time and speeds up product development. Common biomarkers are summarized in Table 10.

Biosensors integrated with the wound dressing have several advantages, such as improving wound care treatment, shortening hospitalization time, reducing healthcare costs, and decreasing the frequency of wound dressing exchange [192]. They solve many challenges associated with wound healing, especially with chronic wounds, by allowing real-time sensing, and responding to and reposting information on the wound environment. Biosensors integrated with wound dressings have the property of proportional flexibility to the hydrogel matrix and body contours, biocompatibility, and non-toxicity. They have the ability to respond to potential infections or hyper-inflammation in chronic wounds [193]. The degradation rate of biosensors has to be proportional to the degradation of the hydrogel matrix, and non-toxic [193]. Temperature can be taken as an early predictor of infection before any other symptom emergence [194]. Several sensors can be integrated with the wound dressings; however, an appropriate design is needed, and an extra cost is added. Hydrogels can respond to changes in temperature, humidity, pH, metal ions, and gases [194,195,196,197]. Moreover, they can detect biomarkers, such as lactate, glucose, and proteins [195,196,197,198,199]. In this review, the most common sensors integrated with wound dressings, i.e., temperature and pH, are discussed.

### 7.1. Temperature Sensor-Integrated Wound Dressings 

**Figure 7:**
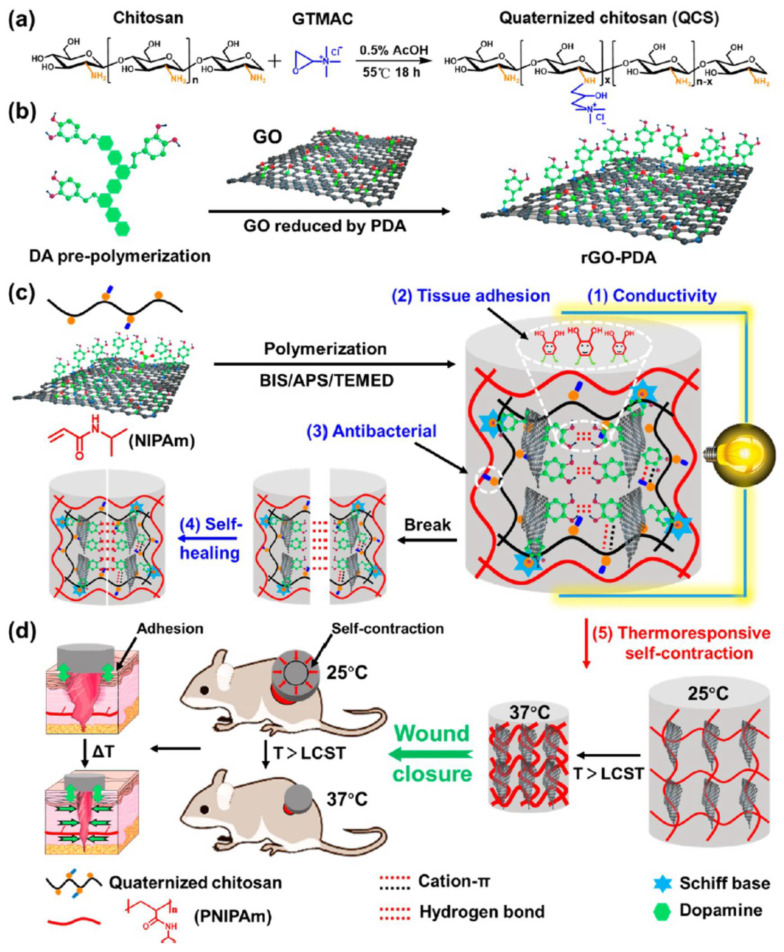
(**a**) Synthesis of the quaternized chitosan (QCS), (**b**) synthesis of the reduction graphene oxide coated by polydopamine (rGO-PDA), (**c**) schematic for the preparation process of the QCS/rGO-PDA/PNIPAm hydrogel and its properties: (1) conductivity, (2) tissue adhesion, (3) antibacterial activity, (4) self-healing, and (5) thermo-responsive self-contraction. (**d**) schematic showing the wound closure by assisting the thermos-responsive hydrogel (QCS/rGO-PDA/PNIPAm) [202].

In recent decades, thermo-reversible hydrogels with temperature-triggered sol-gel transition have shown promising outcomes in drug delivery applications. The temperature-sensitive hydrogels (thermo-gel) are hydrophobic due to the presence of methyl, ethyl, and propyl groups which bind via hydrogen bonds. The most popular thermo-responsive polymer is poly(N-isopropyl acrylamide) (PNIPAAm) [200]. It forms a gel at body temperature (37 °C) due to its low critical solution temperature (LCST) which is 32 °C. To expand its applications in healthcare, its LCST can be modified to be 37 °C through a co-polymerization process. Networks made of hybrid PNIPAAm were demonstrated to have high potential wound closure applications. For instance, PNIPAAm was co-polymerized with alginate and silver nanoparticles for developing wound dressings. The hydrogel dressings generated sufficient temperature to activate wound contraction in order to promote the healing process. Li et al. co-polymerized PNIPAAm with chitosan and polydopamine-coated graphene oxide, to develop multifunctional wound dressings (Figure 7a–c). The PNIPAAm assisted in temperature-dependent drug release [201]. The developed dressings were found to have good thermo-responsive self-contraction and skin adhesion properties. The self-contraction property helped wound closure by actively contracting the wound (Figure 7d). Recently, PNIPAA was co-polymerized with alginate (ALG) and methylcellulose. The gel precursor was 3D printed to develop wound dressings [202]. The wound dressing was loaded with Octenisept^®^, to provide antimicrobial properties. The dressings demonstrated multifunctionality; the PNIPAAm in the dressing assisted in temperature-induced shrinkage to accelerate wound contraction, Octenisept^®^ provided antimicrobial activity, and the incorporated PEGDA enhanced biocompatibility. 

### 7.2. pH Sensor-Integrated Wound Dressings 

When skin is exposed to an injury, the imperceptibly acidic behavior of the skin changes to alkaline. Therefore, trailing the healing of the wound via pH sensors is essential. A pH-sensitive hydrogel can alter its volume in response to a change in the environment’s pH. Hydrogels have a wide function window for pH measurements. Any pH-sensitive hydrogel contains acidic groups, such as carboxylic and sulfonic acids, or basic groups, such as ammonium salts, which respond to pH changes by gaining or losing protons [203]. The main reason for reading the pH ranges of the skin is to monitor the healing rate of skin wounds. Healthy skin has an acidic pH range of 4–6 resulting from the excreted fatty acids from the skin’s sebaceous glands which mix with lactic and amino acids from sweat to create an acidic pH. The skin’s acidity makes it capable of defending against external pathogens. When the skin suffers from an injury or wound, the pH range instantly changes to basic due to fluidic mixing with the body’s internal fluid. The pH level of a wound was found to change while it is healing. As the wound heals, its chronic environment progresses from alkaline conditions to neutral, and finally to acidic conditions [204]. To monitor the volumetric response of pH-responsive hydrogels, transducers are used. The transducers track the changes on two principles: mechanical work which is induced by the change of the volume that can be tracked using a microcantilever bending plate transducer; and observation of changes in properties, such as optical, conductance, and oscillation mechanisms. Immersing the hydrogel inside different pH solutions leads to change in the quantity of dissociated carboxylic ions that alter the volume and refractive index of the hydrogel [205]. Generally, upon swelling of the hydrogels, the refractive index undergoes a negative shift as pure water usually possesses a lower refractive index than the hydrogel in its unhydrated condition [206]. Tomar et al. studied the swelling of pH-responsive particles in response to several pH ranges, and observed that at pH 7.4, there was water uptake [206]. El-Nahhal et al. reported phenol red, a pH indicator, entrapment inside a hydrogel matrix [207]. Phenol red (PR) was trapped into different silica hydrogels in the presence of ethanediyl-1, 2-bis (dimethyldodecylammonium bromide (Gemini 12-2-12), alkyl hydroxyethyl dimethyl ammonium chloride (HY, *R*= 12–14), and sodium dodecyl sulfate (SDS) surfactants [208]. The colorimetric appearance of the hydrogel changed with pH and the absorption peak positions were correlated to the pH values. Liu et al. used another colorimetric pH indicator, red cabbage, to detect wound pH changes [208]. Generally, the cabbage pigment has red color in acidic conditions and turns bluish green in alkaline (basic) solution. The color of the hydrogel patches underwent a transition from yellow at pH of 5, 6, and 7, to orange at pH of 7.4 and 8, and, finally, to red at a pH of 9 [208]. This range of color changes matches the clinically meaningful pH range of chronic or infected wounds. So, if the hydrogel patch is transparent, then the pH level can be witnessed by the naked eye. However, the hydrogel water content and the amount of added calcium could significantly impact the Young’s modulus of the pH-responsive hydrogel. 

**Figure 8:**
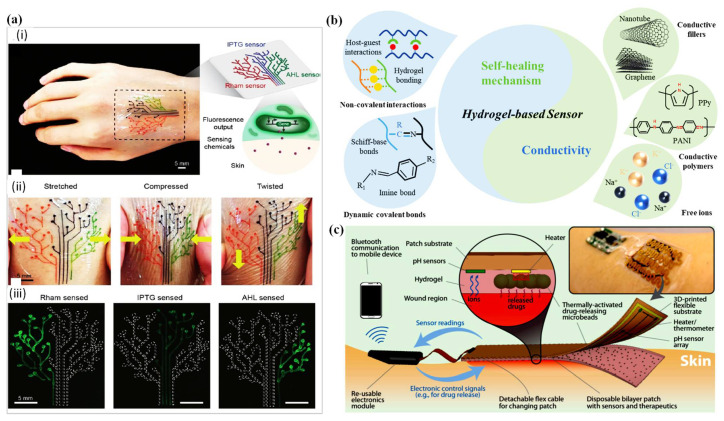
(**a**) Electronic sensors integrated with hydrogels [174], (**b**) the general strategy of hydrogel-based sensors, and (**c**) wearable sensor with conductive self-healing hydrogels [209].

One of the advantages of wound bandage-integrated sensors is the ability to provide reliable information on wound status in real-time continuously, without need to touch the bandage [209]. For instance, a smart patch-integrated pH sensors was developed to monitor wound status using a smartphone as a reader (Figure 8). The sensors were distributed under the hydrogel substrate with microchips that tracked the ions and release drugs. When hydrogels are reinforced with nanomaterial, they show superior properties and tailored functionality [210]. Different sensors can be embedded in hydrogel wound dressings to provide real-time information about wound conditions. However, the dressings must meet certain specifications: (i) the dressing should resemble the wearable sensors that adhere to its mechanical properties, (ii) the dressing should move with the skin’s stretching, compression, and twisting, (iii) the patch should deform relative to the skin healing rate and should not restrain the skin from clotting. Table 11 summarize some examples of 3D printed dressing-integrated sensors and their characteristics.

## 8. Fundamental Challenges and Future Outcomes

The currently available wearables for healing wounds or burn-related injuries have their limitations. Most wound patches are not transparent, leave scars, are non-oxygen permeable, and damage skin cells. In addition, traditional patches do not provide information about the wound curing rate or the status of the wound. Moreover, currently available bandages are costly and have safety concerns when used with drugs. Several printed hydrogel-based wound dressings are available on the market. However, they suffer from low reading quality for the wound conditions. Current DLP printed hydrogel bandages suffer from poor mechanical strength and stability so that novel approaches are needed for clinical implementation. Additionally, dressings such as sheet-printed hydrogels do not function on knees and joints for long-periods due to poor adhesion [204]. 

Generally, parts printed by the stereolithographic technique do not have convenient mechanical properties. Another possible challenge for 3D printing technology is resin homogeneity. Sometimes mixing different ratios of resin ingredients may generate nanoparticle or nano-shape bubbles in the resin that could turn into pores after curing, which undermine mechanical properties by generating fatigue in the final hydrogel wound dressing. The challenges mentioned above can be overcome using post UV- and thermal-curing, especially for patches containing nano-pores. Incorporating gluconic acid with hydrogel wound dressings stabilizes the pH ranges that are significant for bacterial protection. For instance, maintaining wound pH in the range of 3.2–4.5 by use of gluconic acid, creates an environment that prevents pathogen growth [225]. In addition, gluconic acid may enhance environmental moisture, and reduce inflammation and infection [225]. Adding nanofiller to the hydrogel makes transporting active agents, such as drugs, feasible by moving in and out of skin via diffusion and active transport. Since nanomaterials have the property of large surface-to-volume ratio, they have been embedded in wound dressings. Adorinni et al. reported carbon nanomaterials which have a high surface area and a hydrophobic nature that can readily non-covalently bind a large number of bioactive compounds for use in drug delivery applications [164]. Di Luca et al. discussed a skin bandage developed using GO and polyacrylamide and polyethylene glycol methacrylate with a drug-release property [226]. The results showed that adding these nanocomposite materials enhanced the specifications of the wound dressings. Carbon nanomaterials have a vast range of applications when blended with smart hydrogels; however, their interaction with biomolecules is still too complex to comprehend. Carbon nanotubes are toxic and should be treated accordingly.

On the other hand, adding some cations enhances the mechanical strength of printed hydrogels. For instance, K^+^ formed rigid and elastic hydrogels and Ca^2+^ produced stiff and brittle hydrogels. The polymeric binder was mixed with selected conventional cation exchange resin, and a filament was prepared using a mini extruder. This methodology has promise in preparing 3D printed cation exchange membranes (CEMs) with a defined structure. Adding calcium ions with graphene oxide showed an excellent response to the smartness of the material making it a candidate for pressure sensing [227]. Calcium ions proved to be helpful for the performance of PVA-based hydrogel dressings where they served as a crosslinker. Silver nanowires and graphene oxide (GO) were developed to create an artificial skin with the ability to sense pressure variations.

## 9. Recommendations and Conclusions

The natural human placenta has a role in wound healing. Some commercially available placentas are monolayer products such as Amnioband^®^ which is an aseptically processed and dehydrated human amnion, and Amnioexcel^®^ which is a dehydrated amniotic membrane allograft. They are commonly used for skin burns and diabetic foot ulcers, or leg ulcers.

The main value of choosing hydrogels for dressing wounds, are their ideal properties reflected in their nontoxicity that can prevent appalling outcomes. They prevent bacterial infection that impairs wound healing and affects healing duration. They provide a fantastic amount of adhesive material to the wound site. Additionally, they maintain the moisture content of the wound which assists in boosting cell migration and proliferation. Hydrogels adjust to the amount of exudates present in damage tissue. Their oxygen permeability allows diffusion of oxygen to the wound bed to accelerate cell activity. The mechanical and physical properties of hydrogel-based wound dressings enable adherence to the structure of the native skin and mimic the biological nature of the wound. Moreover, hydrogels overcome cost challenges. The design of perfect hydrogels for skin wound healing dressings is an ongoing project, with material choice challenges, issues in design methodology, and issues relating to mechanical defects. The most important factors, such as the appropriate shape, structure, porosity, and homogeneity, of hydrogels need to be fulfilled.

## Figures and Tables

**Table 1:** Common types of wounds [52].

Types of Skin Wounds	Caused by
Puncture	Often caused by a sharp or pointed object. It pierces through the skin and can also affect the soft tissue beneath.
Laceration	The skin is cut open, torn, or torn off completely (avulsion). Lacerations can vary in size, shape, depth, and the left flap of skin.
Pressure injury	Lesions are caused by long periods of pressure over a bony part of the body. The hip and heel are common sites for this wound.
Incision	A surgical wound or intentional cut to the skin.
Abrasion	The skin is scraped or rubbed off. Minor abrasions affect only the top layer of skin. Deep abrasions affect deeper layers of the skin tissues and are more likely to leave a scar.
Thermal	Caused by exposure to extreme hot or cold.
Chemical	Caused by exposure to strong acids or bases, such as those found in cleaning products, pool chemicals, or drain cleaners.

**Table 2:** Conventional clinical skin closure.

Types of Skin Closure Strips	Characterized	Ref.
Skin glue	Helps to hold the wound together and allows it to heal. Most of the time, strips are used on the face, arms, legs, and torso. However, the surface areas are clean and dry.	[53]
Sutures	In deep wounds, stitches are applied under the skin to enhance injury closure. The body can absorb these stitches or a physician can remove the stitches from the skin surface.	[54]
Skin grafts	Are used when the skin around the wound is too damaged to heal together. This may happen with pressure sores or after the skin is removed in surgery. Skin grafts take healthy skin from another area of the body. This healthy skin is then placed over the wound.	[55]

**Table 4:** Commercial production of hydrogel wound dressings.

Type of Hydrogel	Cross Linker	Characterization	Properties	Limitation	Commercial Producing Companies	Ref.
Alginate	Natural ionic cross linker	A polysaccharide supports cell production of collagen I, reducing the concentration of proinflammatory cytokines in chronic wounds. Due to the hydrophilic nature, it can absorb a high amount of wound exudate.	Hemostatic effect	-	*Nu-Gel^®^* (Systagenix), *Tegagel^®^* (3M GmbH)), *Algosteril^®^* (4M Medical GmbH), *Curasorb^®^* Alginate (Medtronic), *Sorbsan^®^* (B. Braun Melsungen AG)) *Flexible^®^* (Coloplast AG), *Kendall™* Hydrocolloid Dressing (Medtronic)).	[102,105]
Chitosan	Natural hydrogel	Hemostatic, bacteriostatic, fungistatic properties.	Accelerates healing rate	Dependent on the molecular weight of the macromolecules low elasticity leading to difficulty in producing fibrous wound dressings.	*KytoCel^®^* (MasterCare Medical GmbH), *Chitoderm^®^* plus (Trusetal Verb and stoffwerk GmbH), a chitosan-coated dressing.	[106,107,108,109,110,111,112]
Collagen protein	Natural hydrogel	It is found in ECM, blood vessels, bones and tendons naturally. Collagens of bovine, porcine and avian derivation are common medical products.	High liquid absorbance capability and good mechanical strength. Enhanced vascularization, granulation tissue formation and collagen deposition via fibroblasts, endothelial cells and keratinocytes.	Rapid loss of stability and shape due to enzymatic degradation. Pathogen transmission risk.	*CellerateRX^®^* (Wound Care Innovations LLC), *Regenecare^®^* Wound Gel (MPM Medical Inc.), *Wun’Dres^®^* (Coloplast AG)), *Biobrane^®^* (Smith & Nephew), *CollaSorb^®^* (Paul Hartmann AG), *Fibracol^®^* (Acelity)) *Medifil^®^* (Human Bio Science, Inc.), *Stimulen™* (Southwest Technologies, Inc.))	[113,114,115,116,117,118,119,120]
Collagen	Synthetic	Well-defined chemical structure and precise modified desired material properties.		Limited activity wound healing process.	polyacrylamide/polysaccharide based *FlexiGel^®^* (Smith & Nephew), Poly(ethylene glycol) (PEG)/oakin based *Oakin^®^* hydrogel wound dressing (Amerigel), Polyurethane (PU) based *AquaClear^®^* dressing (Paul Hartmann AG).	[121]

**Table 6:** Double network characteristics.

Double Network (DN)	Characteristics	Ref.
***t*-DN**	*t*-DN gels become more robust than the *c*-DN gels when the second network is loosely cross-linked. *t*-DN gels have a more simple structure than *c*-DN gels.	[125]
***c*-DN**	Interconnection between the two networks through covalent bonds	[125]

**Table 7:** Summary of common characteristics of 3D printing techniques.

3D Printing Methods	Principle	Materials	Accuracy (µm)	Resolution (µm)	Ref.
Digital light processing (DLP)	Photo-curing by a digital projector	Photopolymer and photo-resin	10–25	x: 25 y: 25 z: 20	[129,130,131]
3D Inkjet printing	Extrusion of ink and powder liquid binding	Photo-resin or hydrogel	100	x: 10 y: 10 z: 50	[132,133]
Selective laser sintering (SLS)	Laser-induced sintering of powder particles	Metallic powder, polyamide, PVC	300	x: 50 y: 50 z: 200	[134]
Polyjet	Deposition of the droplets of the photo-curable liquid material and cured	Polymer	10–20	x: 30 y: 30 z: 20	[135,136,137]
Stereolithography (STL)	UV initiated polymerization cross- section by cross-section	Resin (acrylate or epoxy-based with proprietary photoinitiator)	25–150	x: 10 y: 10 z: 15	[138,139,140]
Fused deposition modeling (FDM)	Extrusion of constant filament	ABS, PLA, wax blend, nylon	350	x: 100 y: 100 z: 250	[141,142,143,144,145,146]

**Table 8:** Commercial hydrogel-based wound dressings.

3D Printing Methods	Principle	Materials	Accuracy (µm)	Resolution (µm)	Ref.
Digital light processing (DLP)	Photo-curing by a digital projector	Photopolymer and photo-resin	10–25	x: 25 y: 25 z: 20	[129,130,131]
3D Inkjet printing	Extrusion of ink and powder liquid binding	Photo-resin or hydrogel	100	x: 10 y: 10 z: 50	[132,133]
Selective laser sintering (SLS)	Laser-induced sintering of powder particles	Metallic powder, polyamide, PVC	300	x: 50 y: 50 z: 200	[134]
Polyjet	Deposition of the droplets of the photo-curable liquid material and cured	Polymer	10–20	x: 30 y: 30 z: 20	[136,137,162]
Stereolithography (STL)	UV initiated polymerization cross- section by cross-section	Resin (acrylate or epoxy based with proprietary photoinitiator)	25–150	x: 10 y: 10 z: 15	[138,139,140]
Fused deposition modelling (FDM)	Extrusion of constant filament	ABS, PLA, wax blend, nylon	350	x: 100 y: 100 z: 250	[141,142,143,144,145,146]

**Table 9:** Common nanocomposite hydrogel wound dressings.

Nanocomposites	Hydrogel Resin	Wound Types	Advantages	Challenges	Ref.
Silver nanoparticles (AgNPs)	Chitosan hydrogel	Acute wounds	Self-cleaning and antibacterial properties.	Crosslinking and 3D printing	[180,181,182]
AgNPs	Chitosan and hyaluronic acid	Diabetic foot ulcers	Resisting antibiotic bacteria	Crosslinking and fabrication of the nanomaterial	[183]
AgNPs	Surface-grafted collagen	Acute wounds	Inhibiting of bacterial growth and increase in membrane water absorption	Agglomeration	[184]
TiO_2_	Collagen	In vivo and in vitro excision wounds.	Accelerate healing	Crosslinking and fabrication of the nanomaterial	[185]
Nano ZnO	Chitin hydrogel	Acute and chronic wounds	Enhanced swelling, blood clotting and antibacterial effect. Absorbing large volumes wound exudate. Controlled degradation, enhanced blood clotting and excellent platelet activation.	Fabrication of the nanomaterial	[178]
Nano ZnO	Nitrocellulose	Hard to cover cut wounds	Flexibility, softness, transparency and conformability.	3D printing	[186]
Gelatin oxidized starch nanofibers	*Lawsonia inermis* (henna)	Treating second degree burn	Enhanced fibroblast attachment, proliferation, collagen secretion and antibacterial activity.	3D printing	[187]

**Table 10:** Common biological biomarker.

Types of Biomarkers	Characteristics	Application	Examples	Ref.
Molecular	They have biophysical properties that allow their measurements in biological samples, such as plasma, serum, cerebrospinal fluid, bronchoalveolar lavage, and biopsy.	Blood glucose	Glucose Hemoglobin A1c levels in diabetes, circulating viral load in viral infections, cholesterol, low-density lipoproteins (LDL), and high-density lipoproteins (HDL) levels in cardiovascular disease.	[188]
Histologic	They are obtained from imaging studies.	Grading and staging of cancers	Prostate-specific antigen (PSA) for prostate cancer and fecal occult blood test for colon cancer.	[190]
Radiography	They reflect a biochemical or molecular alteration in cells, tissues, or fluids.	Bone mineral density	Nuchal scan for prenatal screening. Assessing lesion load and brain atrophy for patients with multiple sclerosis.	[189]
Physiologic	They measures of body processes	Blood pressure	Blood flow Electrocardiogram Functional magnetic resonance imaging. Electroencephalography Metabolism positron emission tomography Spectroscopy.	[191]

**Table 11:** Summary of printed biomarkers with their respective characteristics.

Type of Sensors	Methodology	Characteristics Ref.	
Temperature	Thermo-responsive	The temperature sensor provides information about the inflammation level.	[211]
	3D-printed dual hydrogels with symmetric and alternating segmented tubular structures.	Exhibited spatially programmed swelling behavior in response to temperature in an aqueous environment	[212]
	Graphene oxide (GO) to the PNIPAAm-Laponite composite to enhance the temperature responsivity of the hydrogel and to program the shape change.	GO particles are highly responsive to near-infrared light and act as nano-heaters owing to their photothermal properties and their excellent thermal conductivity	[213]
	Multi-temperature responsive hydrogel-based structure based on copolymerization level and the dependent group chain length.	3D printed multi-gel structures with multiple prescribed volume transition temperatures have potential applications in biological systems	[214]
	Double network hydrogels were synthesized using a micellar copolymerization process of hydrophobic n-octadecyl acrylate (C18) and N,Ndimethylacrylamide (DMA) in NaCl aqueous solution.	3D printed thermo-responsive hydrogel film with submillimeter resolution into a capacitor circuit	[154]
pH	pH sensitive dye embedded inside the hydrogel fiber.	Monitor to detect changes in the acidity and basicity of the skin by changing colors. Healing of the skin indicated by acidic color. The potentiometric pH provides information about bacterial infection.	[215]
	Passive (poly (N-isopropylacrylamide) (PNIPAAm)) to active (poly (2-carboxyethyl acrylate) (PCEA)) layers towards environmental pH changes.	The chemical composition of discrete layers resulted in anisotropic swelling behavior. PCEA (upper layer) swelled in high pH values due to deprotonation of the acid groups while PNIPAAm (lower layer) slightly swelled in an acidic pH.	[216]
	Sodium hydrogen carbonate (NaHCO3) vapor as a cross-linker for collagen to provide a homogeneous gelation.	Collagens as a major extracellular matrix protein have several ionizable groups, such as hydroxyl and amine groups in their molecular chains.	[217]
Moisture content	Absorb water due to void imperfections.	Dynamic shape and geometrical expansion, stretching, folding and bending change in response to variations in environmental humidity.	[218,219,220,221]
		Hydrophilic layer expanded in water and forced a shape change as stretching or folding into the structure	[222]
	Origami-inspired structures including polyurethane hydrogel core and polyurethane elastomer skins.	Discrete localized gaps at elastomeric skin were acting active hinges. During the hydration resulted in different complex structures.	[223]
	Composite ink for 3D printing by incorporating cellulose pulp fibers into carboxymethycellulose (CMC) hydrocolloid.	Printed objects underwent reversibly programmed transformation upon hydration and dehydration.	[202]
Upregulation or downregulation of enzyme levels	Modified chitosan functionalized with a fluorogenic substrate	The presence of various types of enzymes can be detected using florigenic or chromogenic substrate. It is highly useful for detection of specific pathogenic bacteria in wound dressing.	[224]

## Data Availability

The data presented in this study are available on request from the corresponding author.

## References

[1] Kresina T., Kaplowitz L., Johnson K. (2016). Human Immunodeficiency Virus Infection in Young Adults: Treatment of Substance Use Disorders as a Priority Component of HIV Prevention, Care and Treatment in Low and Middle Income Countries. Int. J. AIDS Res..

[2] Reportlinker The Global Advanced Wound Care Market Is Projected to Reach USD 12.8 Billion by 2026 from USD 9.4 Billion in 2021, at a CAGR of 6.2%. https://uk.finance.yahoo.com/news/global-advanced-wound-care-market-103200438.html?guccounter=1.

[3] Sen C.K. (2019). Human wounds and its burden: An updated compendium of estimates. Adv. Wound Care.

[4] Guest J.F., Ayoub N., McIlwraith T., Uchegbu I., Gerrish A., Weidlich D., Vowden K., Vowden P. (2017). Health economic burden that different wound types impose on the UK’s National Health Service. Int. Wound J..

[5] Rezvani Ghomi E., Khalili S., Nouri Khorasani S., Esmaeely Neisiany R., Ramakrishna S. (2019). Wound dressings: Current advances and future directions. J. Appl. Polym. Sci..

[6] Jones V., Grey J.E., Harding K.G. (2006). Wound dressings. BMJ.

[7] Qian Y., Shen Y., Deng S., Liu T., Qi F., Lu Z., Liu L., Shao N., Xie J., Ding F. (2019). Dual functional β-peptide polymer-modified resin beads for bacterial killing and endotoxin adsorption. BMC Mater..

[8] Natarajan S., Harini K., Gajula G.P., Sarmento B., Neves-Petersen M.T., Thiagarajan V. (2019). Multifunctional magnetic iron oxide nanoparticles: Diverse synthetic approaches, surface modifications, cytotoxicity towards biomedical and industrial applications. BMC Mater..

[9] Li X., Jiang Y., Wang F., Fan Z., Wang H., Tao C., Wang Z. (2017). Preparation of polyurethane/polyvinyl alcohol hydrogel and its performance enhancement via compositing with silver particles. RSC Adv..

[10] Heyer K., Augustin M., Protz K., Herberger K., Spehr C., Rustenbach S. (2013). Effectiveness of advanced versus conventional wound dressings on healing of chronic wounds: Systematic review and meta-analysis. Dermatology.

[11] Herndon D.N., Barrow R.E., Rutan R.L., Rutan T.C., Desai M.H., Abston S. (1989). A comparison of conservative versus early excision. Therapies in severely burned patients. Ann. Surg..

[12] Schiestl C., Stiefel D., Meuli M. (2010). Giant naevus, giant excision, eleg (i) ant closure? Reconstructive surgery with Integra Artificial Skin^®^ to treat giant congenital melanocytic naevi in children. J. Plast. Reconstr. Aesthetic Surg..

[13] Schiestl C., Neuhaus K., Biedermann T., Böttcher-Haberzeth S., Reichmann E., Meuli M. (2011). Novel treatment for massive lower extremity avulsion injuries in children: Slow, but effective with good cosmesis. Eur. J. Pediatr. Surg..

[14] Böttcher-Haberzeth S., Kapoor S., Meuli M., Neuhaus K., Biedermann T., Reichmann E., Schiestl C. (2011). Osmotic expanders in children: No filling–no control–no problem?. Eur. J. Pediatr. Surg..

[15] Barbour J.R., Schweppe M., Seung-Jun O. (2008). Lower-extremity burn reconstruction in the child. Eur. J. Pediatr. Surg..

[16] Berman B., Viera M.H., Amini S., Huo R., Jones I.S. (2008). Prevention and management of hypertrophic scars and keloids after burns in children. Eur. J. Pediatr. Surg..

[17] Augustine R., Kalarikkal N., Thomas S. (2014). Advancement of wound care from grafts to bioengineered smart skin substitutes. Prog. Biomater..

[18] Pita-López M.L., Fletes-Vargas G., Espinosa-Andrews H., Rodríguez-Rodríguez R. (2021). Physically cross-linked chitosan-based hydrogels for tissue engineering applications: A state-of-the-art review. Eur. Polym. J..

[19] Rodríguez-Rodríguez R., Espinosa-Andrews H., Velasquillo-Martínez C., García-Carvajal Z.Y. (2020). Composite hydrogels based on gelatin, chitosan and polyvinyl alcohol to biomedical applications: A review. Int. J. Polym. Mater. Polym. Biomater..

[20] Agarwal R., Malhotra S., Gupta V., Jain V. (2022). The application of Three-dimensional printing on foot fractures and deformities: A mini-review. Ann. 3d Print. Med..

[21] Li J., Wu C., Chu P.K., Gelinsky M. (2020). 3D printing of hydrogels: Rational design strategies and emerging biomedical applications. Mater. Sci. Eng. R Rep..

[22] Rajabi M., McConnell M., Cabral J., Ali M.A. (2021). Chitosan hydrogels in 3D printing for biomedical applications. Carbohydr. Polym..

[23] Soleymani Eil Bakhtiari S., Bakhsheshi-Rad H.R., Karbasi S., Razzaghi M., Tavakoli M., Ismail A.F., Sharif S., RamaKrishna S., Chen X., Berto F. (2021). 3-Dimensional Printing of Hydrogel-Based Nanocomposites: A Comprehensive Review on the Technology Description, Properties, and Applications. Adv. Eng. Mater..

[24] Zamboulis A., Michailidou G., Koumentakou I., Bikiaris D.N. (2022). Polysaccharide 3D Printing for Drug Delivery Applications. Pharmaceutics.

[25] Agarwala S. (2020). Electrically conducting hydrogels for health care: Concept, fabrication methods, and applications. Int. J. Bioprinting.

[26] Athukorala S.S., Tran T.S., Balu R., Truong V.K., Chapman J., Dutta N.K., Roy Choudhury N. (2021). 3D printable electrically conductive hydrogel scaffolds for biomedical applications: A review. Polymers.

[27] Advincula R.C., Dizon J.R.C., Caldona E.B., Viers R.A., Siacor F.D.C., Maalihan R.D., Espera A.H. (2021). On the progress of 3D-printed hydrogels for tissue engineering. MRS Commun..

[28] Siree G.K., M Amulya T., M Pramod Kumar T., Sowmya S., Divith K., G Ramu B., P Gowrav M. (2021). Transfiguring Healthcare: Three-Dimensional Printing in Pharmaceutical Sciences; Trends during COVID-19: A Review. J. Pharm. Res. Int..

[29] Topuz M., Dikici B., Gavgali M., Yilmazer H. (2018). A review on the hydrogels used in 3D Bio-printing. Int. J. 3D Print. Technol. Digital Ind..

[30] Paul G.M., Rezaienia A., Wen P., Condoor S., Parkar N., King W., Korakianitis T. (2018). Medical applications for 3D printing: Recent developments. Mo. Med..

[31] Fayyazbakhsh F., Leu M.C. (2020). A brief review on 3D bioprinted skin substitutes. Procedia Manuf..

[32] de Oliveira R.S., Fantaus S.S., Guillot A.J., Melero A., Beck R.C.R. (2021). 3D-Printed Products for Topical Skin Applications: From Personalized Dressings to Drug Delivery. Pharmaceutics.

[33] Sun W., Schaffer S., Dai K., Yao L., Feinberg A., Webster-Wood V. (2021). 3D Printing Hydrogel-Based Soft and Biohybrid Actuators: A Mini-Review on Fabrication Techniques, Applications, and Challenges. Front. Robot. AI.

[34] Li N., Qiao D., Zhao S., Lin Q., Zhang B., Xie F. (2021). 3D printing to innovate biopolymer materials for demanding applications: A review. Mater. Today Chem..

[35] Beheshtizadeh N., Lotfibakhshaiesh N., Pazhouhnia Z., Hoseinpour M., Nafari M. (2020). A review of 3D bio-printing for bone and skin tissue engineering: A commercial approach. J. Mater. Sci..

[36] Waghmare V.S., Wadke P.R., Dyawanapelly S., Deshpande A., Jain R., Dandekar P. (2018). Starch based nanofibrous scaffolds for wound healing applications. Bioact. Mater..

[37] Calonge W.M., AlAli A.B., Griffin M., Butler P.E. (2016). Three-dimensional printing of models of cleft lip and palate. Plast. Reconstr. Surg. Glob. Open.

[38] Askari M., Naniz M.A., Kouhi M., Saberi A., Zolfagharian A., Bodaghi M. (2021). Recent progress in extrusion 3D bioprinting of hydrogel biomaterials for tissue regeneration: A comprehensive review with focus on advanced fabrication techniques. Biomater. Sci..

[39] Ghosh S., Kaushik G., Roy P., Lahiri D. (2021). Application of 3D Bioprinting in Wound Healing: A Review. Trends Biomater. Artif. Organs.

[40] Gupta S., Bissoyi A., Bit A. (2018). A review on 3D printable techniques for tissue engineering. BioNanoScience.

[41] He P., Zhao J., Zhang J., Li B., Gou Z., Gou M., Li X. (2018). Bioprinting of skin constructs for wound healing. Burn. Trauma.

[42] Li H., Tan C., Li L. (2018). Review of 3D printable hydrogels and constructs. Mater. Des..

[43] Li X., Cui R., Sun L., Aifantis K.E., Fan Y., Feng Q., Cui F., Watari F. (2014). 3D-printed biopolymers for tissue engineering application. Int. J. Polym. Sci..

[44] Sahranavard M., Zamanian A., Ghorbani F., Shahrezaee M.H. (2020). A critical review on three dimensional-printed chitosan hydrogels for development of tissue engineering. Bioprinting.

[45] Malekmohammadi S., Sedghi Aminabad N., Sabzi A., Zarebkohan A., Razavi M., Vosough M., Bodaghi M., Maleki H. (2021). Smart and Biomimetic 3D and 4D Printed Composite Hydrogels: Opportunities for Different Biomedical Applications. Biomedicines.

[46] Smandri A., Nordin A., Hwei N.M., Chin K.-Y., Abd Aziz I., Fauzi M.B. (2020). Natural 3D-printed bioinks for skin regeneration and wound healing: A systematic review. Polymers.

[47] Nadhif M.H., Assyarify H., Irsyad M., Pramesti A.R., Suhaeri M. (2021). Recent advances in 3D printed wound dressings. AIP Conference Proceedings.

[48] Vig K., Chaudhari A., Tripathi S., Dixit S., Sahu R., Pillai S., Dennis V.A., Singh S.R. (2017). Advances in skin regeneration using tissue engineering. Int. J. Mol. Sci..

[49] Metcalfe A.D., Ferguson M.W. (2007). Tissue engineering of replacement skin: The crossroads of biomaterials, wound healing, embryonic development, stem cells and regeneration. J. R. Soc Interface.

[50] Pereira R.F., Sousa A., Barrias C.C., Bayat A., Granja P.L., Bártolo P.J. (2017). Advances in bioprinted cell-laden hydrogels for skin tissue engineering. Biomanufacturing Rev..

[51] Uccioli L., Izzo V., Meloni M., Vainieri E., Ruotolo V., Giurato L. (2015). Non-healing foot ulcers in diabetic patients: General and local interfering conditions and management options with advanced wound dressings. J. Wound Care.

[52] Dorishetty P., Dutta N.K., Choudhury N.R. (2020). Bioprintable tough hydrogels for tissue engineering applications. Adv. Colloid Interface Sci..

[53] Streitz M.J. How To Cleanse, Irrigate, Debride, and Dress Wounds. https://www.msdmanuals.com/professional/injuries-poisoning/how-to-care-for-wounds-and-lacerations/how-to-cleanse,-irrigate,-debride,-and-dress-wounds.

[54] Gottrup F. (1999). Wound closure techniques. J. Wound Care.

[55] Andreassi A., Bilenchi R., Biagioli M., D’Aniello C. (2005). Classification and pathophysiology of skin grafts. Clin. Dermatol..

[56] Bielefeld K.A., Amini-Nik S., Alman B.A. (2013). Cutaneous wound healing: Recruiting developmental pathways for regeneration. Cell. Mol. Life Sci..

[57] Li W.-H., Pappas A., Zhang L., Ruvolo E., Cavender D. (2013). IL-11, IL-1α, IL-6, and TNF-α are induced by solar radiation in vitro and may be involved in facial subcutaneous fat loss in vivo. J. Dermatol. Sci..

[58] Greaves N.S., Ashcroft K.J., Baguneid M., Bayat A. (2013). Current understanding of molecular and cellular mechanisms in fibroplasia and angiogenesis during acute wound healing. J. Dermatol. Sci..

[59] FrykbergRobert G. (2015). Challenges in the treatment of chronic wounds. Adv. Wound Care.

[60] Chouhan D., Chakraborty B., Nandi S.K., Mandal B.B. (2017). Role of non-mulberry silk fibroin in deposition and regulation of extracellular matrix towards accelerated wound healing. Acta Biomater..

[61] Zahedi P., Rezaeian I., Ranaei-Siadat S.O., Jafari S.H., Supaphol P. (2010). A review on wound dressings with an emphasis on electrospun nanofibrous polymeric bandages. Polym. Adv. Technol..

[62] Percival N.J. (2002). Classification of wounds and their management. Surgery (Oxford).

[63] Hadian Y., Bagood M.D., Dahle S.E., Sood A., Isseroff R.R. (2019). Interleukin-17: Potential target for chronic wounds. Mediat. Inflamm..

[64] Golinko M.S., Clark S., Rennert R., Flattau A., Boulton A.J., Brem H. (2009). Wound emergencies: The importance of assessment, documentation, and early treatment using a wound electronic medical record. Ostomy/Wound Manag..

[65] Moore K., McCallion R., Searle R.J., Stacey M.C., Harding K.G. (2006). Prediction and monitoring the therapeutic response of chronic dermal wounds. Int. Wound J..

[66] Chandika P., Ko S.-C., Jung W.-K. (2015). Marine-derived biological macromolecule-based biomaterials for wound healing and skin tissue regeneration. Int. J. Biol. Macromol..

[67] Laiva A.L., O’Brien F.J., Keogh M.B. (2018). Innovations in gene and growth factor delivery systems for diabetic wound healing. J. Tissue Eng. Regen. Med..

[68] Morton L.M., Phillips T.J. (2016). Wound healing and treating wounds: Differential diagnosis and evaluation of chronic wounds. J. Am. Acad. Dermatol..

[69] Yee A., Patel M., Wu E., Yi S., Marti G., Harmon J. IDr: An Intelligent Digital Ruler App for Remote Wound Assessment. Proceedings of the 2016 IEEE First International Conference on Connected Health: Applications, Systems and Engineering Technologies (CHASE).

[70] Trengove N.J., Stacey M.C., Macauley S., Bennett N., Gibson J., Burslem F., Murphy G., Schultz G. (1999). Analysis of the acute and chronic wound environments: The role of proteases and their inhibitors. Wound Repair Regen..

[71] Rossaint J., Margraf A., Zarbock A. (2018). Role of platelets in leukocyte recruitment and resolution of inflammation. Front. Immunol..

[72] Versteeg H., Heemskerk J., Levi M., Reitsma P. (2013). New fundamentals in coagulation. Physiol. Rev..

[73] Schultz G.S., Chin G.A., Moldawer L., Diegelmann R.F., Fitridge R T.M. (2011). 23 Principles of Wound Healing. Mechanisms of Vascular Disease: A Reference Book for Vascular Specialists.

[74] Midwood K.S., Williams L.V., Schwarzbauer J.E. (2004). Tissue repair and the dynamics of the extracellular matrix. Int. J. Biochem. Cell Biol..

[75] Desjardins-Park H.E., Foster D.S., Longaker M.T. (2018). Fibroblasts and wound healing: An update. Regerative Med..

[76] Bowden L., Byrne H., Maini P., Moulton D. (2016). A morphoelastic model for dermal wound closure. Biomech. Model. Mechanobiol..

[77] Yee A., Harmon J., Yi S. (2016). Quantitative monitoring wound healing status through three-dimensional imaging on mobile platforms. J. Am. Coll. Clin. Wound Spéc..

[78] Bahram M., Mohseni N., Moghtader M. (2016). An introduction to hydrogels and some recent applications. Emerging Concepts in Analysis and Applications of Hydrogels.

[79] Darge H.F., Andrgie A.T., Tsai H.-C., Lai J.-Y. (2019). Polysaccharide and polypeptide based injectable thermo-sensitive hydrogels for local biomedical applications. Int. J. Biol. Macromol..

[80] Dissemond J., Augustin M., Eming S.A., Goerge T., Horn T., Karrer S., Schumann H., Stücker M., for the working group for wound healing (AGW) of the German Society of Dermatology (DDG) (2014). Modern wound care–practical aspects of non-interventional topical treatment of patients with chronic wounds. J. Der Dtsch. Dermatol. Ges..

[81] Powers J.G., Morton L.M., Phillips T.J. (2013). Dressings for chronic wounds. Dermatol. Ther..

[82] Skórkowska-Telichowska K., Czemplik M., Kulma A., Szopa J. (2013). The local treatment and available dressings designed for chronic wounds. J. Am. Acad. Dermatol..

[83] Chen C., Liu L., Huang T., Wang Q. (2013). Bubble template fabrication of chitosan/poly (vinyl alcohol) sponges for wound dressing applications. Int. J. Biol. Macromol..

[84] Fan L., Yang J., Wu H., Hu Z., Yi J., Tong J., Zhu X. (2015). Preparation and characterization of quaternary ammonium chitosan hydrogel with significant antibacterial activity. Int. J. Biol. Macromol..

[85] Khodja A.N., Mahlous M., Tahtat D., Benamer S., Youcef S.L., Chader H., Mouhoub L., Sedgelmaci M., Ammi N., Mansouri M.B. (2013). Evaluation of healing activity of PVA/chitosan hydrogels on deep second degree burn: Pharmacological and toxicological tests. Burns.

[86] Anjum S., Arora A., Alam M., Gupta B. (2016). Development of antimicrobial and scar preventive chitosan hydrogel wound dressings. Int. J. Pharm..

[87] Chen S.-H., Tsao C.-T., Chang C.-H., Lai Y.-T., Wu M.-F., Chuang C.-N., Chou H.-C., Wang C.-K., Hsieh K.-H. (2013). Assessment of reinforced poly (ethylene glycol) chitosan hydrogels as dressings in a mouse skin wound defect model. Mater. Sci. Eng. C.

[88] Aïssa B., Therriault D., Haddad E., Jamroz W. (2012). Self-healing materials systems: Overview of major approaches and recent developed technologies. Adv. Mater. Sci. Eng..

[89] Arnold R.M., Huddleston N.E., Locklin J. (2012). Utilizing click chemistry to design functional interfaces through post-polymerization modification. J. Mater. Chem..

[90] Billiet S., Hillewaere X.K., Teixeira R.F., Du Prez F.E. (2013). Chemistry of crosslinking processes for self-healing polymers. Macromol. Rapid Commun..

[91] Choi S.W., Zhang Y., Xia Y. (2010). A temperature-sensitive drug release system based on phase-change materials. Angew. Chem. Int. Ed..

[92] Devi N., Kakati D.K. (2013). Smart porous microparticles based on gelatin/sodium alginate polyelectrolyte complex. J. Food Eng..

[93] De La Rica R., Aili D., Stevens M.M. (2012). Enzyme-responsive nanoparticles for drug release and diagnostics. Adv. Drug Deliv. Rev..

[94] Kokabi M., Sirousazar M., Hassan Z.M. (2007). PVA–clay nanocomposite hydrogels for wound dressing. Eur. Polym. J..

[95] Kamoun E.A., Kenawy E.-R.S., Chen X. (2017). A review on polymeric hydrogel membranes for wound dressing applications: PVA-based hydrogel dressings. J. Adv. Res..

[96] Banerjee I., Mishra D., Das T., Maiti T.K. (2012). Wound pH-responsive sustained release of therapeutics from a poly (NIPAAm-co-AAc) hydrogel. J. Biomater. Science. Polym. Ed..

[97] Francesko A., Petkova P., Tzanov T. (2018). Hydrogel dressings for advanced wound management. Curr. Med. Chem..

[98] Cutting K.F. (2003). Wound exudate: Composition and functions. Br. J. Community Nurs..

[99] Liang M., Chen Z., Wang F., Liu L., Wei R., Zhang M. (2019). Preparation of self-regulating/anti-adhesive hydrogels and their ability to promote healing in burn wounds. J. Biomed. Mater. Res. Part B Appl. Biomater..

[100] Sezer A.D., Cevher E. (2011). Biopolymers as wound healing materials: Challenges and new strategies. Biomater. Appl. Nanomed..

[101] Xue M., Zhao R., Lin H., Jackson C. (2018). Delivery systems of current biologicals for the treatment of chronic cutaneous wounds and severe burns. Adv. Drug Deliv. Rev..

[102] Miguel S.P., Moreira A.F., Correia I.J. (2019). Chitosan based-asymmetric membranes for wound healing: A review. Int. J. Biol. Macromol..

[103] Weller C., Team V. (2019). Interactive dressings and their role in moist wound management. Advanced Textiles for Wound Care.

[104] Op’t Veld R.C., Walboomers X.F., Jansen J.A., Wagener F.A. (2020). Design considerations for hydrogel wound dressings: Strategic and molecular advances. Tissue Eng. Part B Rev..

[105] Wiegand C., Heinze T., Hipler U.C. (2009). Comparative in vitro study on cytotoxicity, antimicrobial activity, and binding capacity for pathophysiological factors in chronic wounds of alginate and silver-containing alginate. Wound Repair Regen..

[106] Ducheyne P., Healy K.E., Hutmacher D.W., Grainger D.W., Kirkpatrick C.J. (2015). Comprehensive Biomaterials.

[107] Jayakumar R., Prabaharan M., Kumar P.S., Nair S., Tamura H. (2011). Biomaterials based on chitin and chitosan in wound dressing applications. Biotechnol. Adv..

[108] Moura L.I., Dias A.M., Leal E.C., Carvalho L., de Sousa H.C., Carvalho E. (2014). Chitosan-based dressings loaded with neurotensin—an efficient strategy to improve early diabetic wound healing. Acta Biomater..

[109] Phaechamud T., Yodkhum K., Charoenteeraboon J., Tabata Y. (2015). Chitosan–aluminum monostearate composite sponge dressing containing asiaticoside for wound healing and angiogenesis promotion in chronic wound. Mater. Sci. Eng. C.

[110] Patrulea V., Ostafe V., Borchard G., Jordan O. (2015). Chitosan as a starting material for wound healing applications. Eur. J. Pharm. Biopharm..

[111] Zheng L.-Y., Zhu J.-F. (2003). Study on antimicrobial activity of chitosan with different molecular weights. Carbohydr. Polym..

[112] Kean T., Thanou M. (2010). Biodegradation, biodistribution and toxicity of chitosan. Adv. Drug Deliv. Rev..

[113] Ågren M. (2016). Wound Healing Biomaterials-Volume 2: Functional Biomaterials.

[114] Mogoşanu G.D., Grumezescu A.M. (2014). Natural and synthetic polymers for wounds and burns dressing. Int. J. Pharm..

[115] Koehler J., Brandl F.P., Goepferich A.M. (2018). Hydrogel wound dressings for bioactive treatment of acute and chronic wounds. Eur. Polym. J..

[116] Chattopadhyay S., Raines R.T. (2014). Collagen-based biomaterials for wound healing. Biopolymers.

[117] Moura L.I., Dias A.M., Suesca E., Casadiegos S., Leal E.C., Fontanilla M.R., Carvalho L., de Sousa H.C., Carvalho E. (2014). Neurotensin-loaded collagen dressings reduce inflammation and improve wound healing in diabetic mice. Biochim. Biophys. Acta.

[118] Helary C., Abed A., Mosser G., Louedec L., Letourneur D., Coradin T., Giraud-Guille M.M., Meddahi-Pellé A. (2015). Evaluation of dense collagen matrices as medicated wound dressing for the treatment of cutaneous chronic wounds. Biomater. Sci..

[119] Willard J.J., Drexler J.W., Das A., Roy S., Shilo S., Shoseyov O., Powell H.M. (2013). Plant-derived human collagen scaffolds for skin tissue engineering. Tissue Eng. Part A.

[120] Natarajan V., Krithica N., Madhan B., Sehgal P.K. (2013). Preparation and properties of tannic acid cross-linked collagen scaffold and its application in wound healing. J. Biomed. Mater. Res. Part B Appl. Biomater..

[121] Mayet N., Choonara Y.E., Kumar P., Tomar L.K., Tyagi C., Du Toit L.C., Pillay V. (2014). A comprehensive review of advanced biopolymeric wound healing systems. J. Pharm. Sci..

[122] Arora G., Singh I., Nagpal M., Arora S. (2011). Recent advances in stimuli induced pulsatile drug delivery system: A review. Res. J. Pharm. Technol..

[123] Gong J.P., Katsuyama Y., Kurokawa T., Osada Y. (2003). Double-network hydrogels with extremely high mechanical strength. Adv. Mater..

[124] Smith D., Seifi D.L.B.W.F.H.K.M. (2020). Vat Polymerization. Additive Manufacturing Process.

[125] Das N. (2013). Preparation methods and properties of hydrogel: A review. Int. J. Pharm. Pharm. Sci..

[126] Joshi S., Chang T.-C. (1988). Graph-based heuristics for recognition of machined features from a 3D solid model. Comput. Des..

[127] Dori D., Tombre K. (1995). From engineering drawings to 3D CAD models: Are we ready now?. Comput. Des..

[128] Tack P., Victor J., Gemmel P., Annemans L. (2016). 3D-printing techniques in a medical setting: A systematic literature review. Biomed. Eng. Online.

[129] Tiller B., Reid A., Zhu B., Guerreiro J., Domingo-Roca R., Jackson J.C., Windmill J. (2019). Piezoelectric microphone via a digital light processing 3D printing process. Mater. Des..

[130] Ge L., Dong L., Wang D., Ge Q., Gu G. (2018). A digital light processing 3D printer for fast and high-precision fabrication of soft pneumatic actuators. Sens. Actuators A Phys..

[131] Sharafeldin M., Jones A., Rusling J.F. (2018). 3D-printed biosensor arrays for medical diagnostics. Micromachines.

[132] Mannoor M.S., Jiang Z., James T., Kong Y.L., Malatesta K.A., Soboyejo W.O., Verma N., Gracias D.H., McAlpine M.C. (2013). 3D printed bionic ears. Nano Lett..

[133] Low Z.-X., Chua Y.T., Ray B.M., Mattia D., Metcalfe I.S., Patterson D.A. (2017). Perspective on 3D printing of separation membranes and comparison to related unconventional fabrication techniques. J. Membr. Sci..

[134] Zhang J.X., Hoshino K. (2018). Molecular Sensors and Nanodevices: Principles, Designs and Applications in Biomedical Engineering.

[135] Jammalamadaka U., Tappa K. (2018). Recent advances in biomaterials for 3D printing and tissue engineering. J. Funct. Biomater..

[136] Rusling J.F. (2018). Developing microfluidic sensing devices using 3D printing. ACS Sens..

[137] Wang K., Ho C.-C., Zhang C., Wang B. (2017). A review on the 3D printing of functional structures for medical phantoms and regenerated tissue and organ applications. Engineering.

[138] Valentin T.M., Leggett S.E., Chen P.-Y., Sodhi J.K., Stephens L.H., McClintock H.D., Sim J.Y., Wong I.Y. (2017). Stereolithographic printing of ionically-crosslinked alginate hydrogels for degradable biomaterials and microfluidics. Lab. Chip.

[139] Hinman S.S., McKeating K.S., Cheng Q. (2017). Plasmonic sensing with 3D printed optics. Anal. Chem..

[140] Wang Z., Kumar H., Tian Z., Jin X., Holzman J.F., Menard F., Kim K. (2018). Visible light photoinitiation of cell-adhesive gelatin methacryloyl hydrogels for stereolithography 3D bioprinting. ACS Appl. Mater. Interfaces.

[141] Rumley-ouellette B.J., Wahry J.H., Baker A.M., Bernardin J.D., Marchi A.N., Todd M.D. In situ printing of conductive poly lactic acid strain sensors embedded into additively manufactured parts. Proceedings of the 11th International Workshop on Structural Health Monitoring.

[142] Cevenini L., Calabretta M.M., Tarantino G., Michelini E., Roda A. (2016). Smartphone-interfaced 3D printed toxicity biosensor integrating bioluminescent “sentinel cells”. Sens. Actuators B Chem..

[143] Pranzo D., Larizza P., Filippini D., Percoco G. (2018). Extrusion-based 3D printing of microfluidic devices for chemical and biomedical applications: A topical review. Micromachines.

[144] Loo A.H., Chua C.K., Pumera M. (2017). DNA biosensing with 3D printing technology. Analyst.

[145] Song Y., Nesaei S., Du D., Gozen A., Lin Y. 3D Printed Wearable Glucose Sensors. Proceedings of the ECS Meeting Abstracts.

[146] Connell J.L., Kim J., Shear J.B., Bard A.J., Whiteley M. (2014). Real-time monitoring of quorum sensing in 3D-printed bacterial aggregates using scanning electrochemical microscopy. Proc. Natl. Acad. Sci. USA.

[147] Cretu A., Gattin R., Brachais L., Barbier-Baudry D. (2004). Synthesis and degradation of poly (2-hydroxyethyl methacrylate)-graft-poly (ε-caprolactone) copolymers. Polym. Degrad. Stab..

[148] Kim B., Peppas N.A. (2003). Poly (ethylene glycol)-containing hydrogels for oral protein delivery applications. Biomed. Microdevices.

[149] Richter A., Paschew G., Klatt S., Lienig J., Arndt K.-F., Adler H.-J.P. (2008). Review on hydrogel-based pH sensors and microsensors. Sensors.

[150] Zhang Y., Wu F., Li M., Wang E. (2005). pH switching on-off semi-IPN hydrogel based on cross-linked poly (acrylamide-co-acrylic acid) and linear polyallyamine. Polymer.

[151] Jirkovec R., Samkova A., Kalous T., Chaloupek J., Chvojka J. (2021). Preparation of a Hydrogel Nanofiber Wound Dressing. Nanomaterials.

[152] Bourges X., Weiss P., Coudreuse A., Daculsi G., Legeay G. (2002). General properties of silated hydroxyethylcellulose for potential biomedical applications. Biopolym. Orig. Res. Biomol..

[153] Nakajima T., Furukawa H., Tanaka Y., Kurokawa T., Osada Y., Gong J.P. (2009). True chemical structure of double network hydrogels. Macromolecules.

[154] Greenwood J. (2017). The evolution of acute burn care–retiring the split skin graft. Ann. R. Coll. Surg. Engl..

[155] Wong K.V., Hernandez A. (2012). A review of additive manufacturing. Int. Scholarly Res. Not..

[156] Vaezi M., Seitz H., Yang S. (2013). A review on 3D micro-additive manufacturing technologies. Int. J. Adv. Manuf..

[157] McMenamin P.G., Quayle M.R., McHenry C.R., Adams J.W. (2014). The production of anatomical teaching resources using three-dimensional (3D) printing technology. Anat. Sci. Educ..

[158] Lu B., Li D., Tian X. (2015). Development trends in additive manufacturing and 3D printing. Engineering. Epub Ahead Print.

[159] Ventola C.L. (2014). Medical applications for 3D printing: Current and projected uses. Pharm. Ther..

[160] Zhang X., Li Y., He D., Ma Z., Liu K., Xue K., Li H. (2021). An effective strategy for preparing macroporous and self-healing bioactive hydrogels for cell delivery and wound healing. Chem. Eng. J..

[161] Martelli N., Serrano C., van den Brink H., Pineau J., Prognon P., Borget I., El Batti S. (2016). Advantages and disadvantages of 3-dimensional printing in surgery: A systematic review. Surgery.

[162] Tappa K., Jammalamadaka U. (2018). Novel biomaterials used in medical 3D printing techniques. J. Funct. Biomater..

[163] Taormina G., Sciancalepore C., Messori M., Bondioli F. (2018). 3D printing processes for photocurable polymeric materials: Technologies, materials, and future trends. J. Appl. Biomater. Funct. Mater..

[164] Benjamin A.D., Abbasi R., Owens M., Olsen R.J., Walsh D.J., LeFevre T.B., Wilking J.N. (2019). Light-based 3D printing of hydrogels with high-resolution channels. Biomed. Phys. Eng. Express.

[165] Alketbi A.S., Shi Y., Li H., Raza A., Zhang T. (2021). Impact of PEGDA photopolymerization in micro-stereolithography on 3D printed hydrogel structure and swelling. Soft Matter.

[166] Sherman S.L., Kadioglu O., Currier G.F., Kierl J.P., Li J. (2020). Accuracy of digital light processing printing of 3-dimensional dental models. Am. J. Orthod..

[167] Steyrer B., Busetti B., Harakály G., Liska R., Stampfl J. (2018). Hot Lithography vs. room temperature DLP 3D-printing of a dimethacrylate. Addit. Manuf..

[168] Ge Q., Chen Z., Cheng J., Zhang B., Zhang Y.-F., Li H., He X., Yuan C., Liu J., Magdassi S. (2021). 3D printing of highly stretchable hydrogel with diverse UV curable polymers. Sci. Adv..

[169] Cascone S., Lamberti G. (2020). Hydrogel-based commercial products for biomedical applications: A review. Int. J. Pharm..

[170] Schiavon M., Francescon M., Drigo D., Salloum G., Baraziol R., Tesei J., Fraccalanza E., Barbone F. (2016). The use of Integra dermal regeneration template versus flaps for reconstruction of full-thickness scalp defects involving the calvaria: A cost–benefit analysis. Aesthetic Plast. Surg..

[171] Zarybnicka L., Stranska E. (2020). Preparation of cation exchange filament for 3D membrane print. Rapid Prototyp. J..

[172] Ren Y., Yu X., Li Z., Liu D., Xue X. (2020). Fabrication of pH-responsive TA-keratin bio-composited hydrogels encapsulated with photoluminescent GO quantum dots for improved bacterial inhibition and healing efficacy in wound care management: In vivo wound evaluations. J. Photochem. Photobiol. B.

[173] Hou S., Lake R., Park S., Edwards S., Jones C., Jeong K.J. (2018). Injectable macroporous hydrogel formed by enzymatic cross-linking of gelatin microgels. ACS Appl. Bio Mater..

[174] Zhao Y., Chen Y., Zhou Y. (2019). Novel mechanical models of tensile strength and elastic property of FDM AM PLA materials: Experimental and theoretical analyses. Mater. Des..

[175] Hu Y., Wu B., Xiong Y., Tao R., Panayi A.C., Chen L., Tian W., Xue H., Shi L., Zhang X. (2021). Cryogenic 3D printed hydrogel scaffolds loading exosomes accelerate diabetic wound healing. Chem. Eng. J..

[176] Chen H., Cheng R., Zhao X., Zhang Y., Tam A., Yan Y., Shen H., Zhang Y.S., Qi J., Feng Y. (2019). An injectable self-healing coordinative hydrogel with antibacterial and angiogenic properties for diabetic skin wound repair. NPG Asia Mater..

[177] Sudheesh Kumar P., Lakshmanan V.-K., Anilkumar T., Ramya C., Reshmi P., Unnikrishnan A., Nair S.V., Jayakumar R. (2012). Flexible and microporous chitosan hydrogel/nano ZnO composite bandages for wound dressing: In vitro and in vivo evaluation. ACS Appl. Mater. Interfaces.

[178] Kumar P., Lakshmanan V.-K., Biswas R., Nair S.V., Jayakumar R. (2012). Synthesis and biological evaluation of chitin hydrogel/nano ZnO composite bandage as antibacterial wound dressing. J. Biomed. Nanotechnol..

[179] Leppiniemi J., Lahtinen P., Paajanen A., Mahlberg R., Metsä-Kortelainen S., Pinomaa T., Pajari H., Vikholm-Lundin I., Pursula P., Hytönen V.P. (2017). 3D-printable bioactivated nanocellulose–alginate hydrogels. ACS Appl. Mater. Interfaces.

[180] Rivero P.J., Urrutia A., Goicoechea J., Arregui F.J. (2015). Nanomaterials for functional textiles and fibers. Nanoscale Res. Lett..

[181] Montazer M., Seifollahzadeh S. (2011). Enhanced self-cleaning, antibacterial and UV protection properties of nano TiO2 treated textile through enzymatic pretreatment. Photochem. Photobiol..

[182] Gao L., Gan H., Meng Z., Gu R., Wu Z., Zhu X., Sun W., Li J., Zheng Y., Sun T. (2016). Evaluation of genipin-crosslinked chitosan hydrogels as a potential carrier for silver sulfadiazine nanocrystals. Colloids Surf. B. Biointerfaces.

[183] Anisha B., Biswas R., Chennazhi K., Jayakumar R. (2013). Chitosan–hyaluronic acid/nano silver composite sponges for drug resistant bacteria infected diabetic wounds. Int. J. Biol. Macromol..

[184] Bao J., Yang B., Sun Y., Zu Y., Deng Y. (2013). A berberine-loaded electrospun poly-(ε-caprolactone) nanofibrous membrane with hemostatic potential and antimicrobial property for wound dressing. J. Biomed. Nanotechnol..

[185] Archana D., Singh B.K., Dutta J., Dutta P. (2013). In vivo evaluation of chitosan–PVP–titanium dioxide nanocomposite as wound dressing material. Carbohydr. Polym..

[186] Mu X., Yu H., Zhang C., Chen X., Cheng Z., Bai R., Wu X., Yu Q., Wu C., Diao Y. (2016). Nano-porous nitrocellulose liquid bandage modulates cell and cytokine response and accelerates cutaneous wound healing in a mouse model. Carbohydr. Polym..

[187] Hadisi Z., Nourmohammadi J., Nassiri S.M. (2018). The antibacterial and anti-inflammatory investigation of Lawsonia Inermis-gelatin-starch nano-fibrous dressing in burn wound. Int. J. Biol. Macromol..

[188] Oyejide L., Mendes O.R., Mikaelian I. (2017). Molecular Pathology: Applications in Nonclinical Drug Development. A Comprehensive Guide to Toxicology in Nonclinical Drug Development.

[189] Lou E., Johnson M., Sima C., Gonzalez-Espinoza R., Fleisher M., Kris M.G., Azzoli C.G. (2014). Serum biomarkers for assessing histology and outcomes in patients with metastatic lung cancer. Cancer Biomark..

[190] Newman J.S., Bree R.L., Rubin J.M. (1995). Prostate cancer: Diagnosis with color Doppler sonography with histologic correlation of each biopsy site. Radiology.

[191] Waghmare L.S., Srivastava T.K. (2016). Conceptualizing physiology of arterial blood pressure regulation through the logic model. Adv. Physiol. Educ..

[192] Myers S., Navsaria H., Ojeh N. (2014). Skin engineering and keratinocyte stem cell therapy. Tissue Eng..

[193] Shakespeare P.G. (2005). The role of skin substitutes in the treatment of burn injuries. Clin. Dermatol..

[194] Stiefel D., Schiestl C., Meuli M. (2010). Integra Artificial Skin^®^ for burn scar revision in adolescents and children. Burns.

[195] Broussard K.C., Powers J.G. (2013). Wound dressings: Selecting the most appropriate type. Am. J. Clin. Dermatol..

[196] Yao M., Attalla K., Ren Y., French M.A., Driver V.R. (2013). Ease of use, safety, and efficacy of integra bilayer wound matrix in the treatment of diabetic foot ulcers in an outpatient clinical setting: A prospective pilot study. J. Am. Podiatr. Med. Assoc..

[197] Böttcher-Haberzeth S., Biedermann T., Schiestl C., Hartmann-Fritsch F., Schneider J., Reichmann E., Meuli M. (2012). Matriderm^®^ 1 mm versus Integra^®^ Single Layer 1.3 mm for one-step closure of full thickness skin defects: A comparative experimental study in rats. Pediatric Surg. Int..

[198] Kolokythas P., Aust M., Vogt P., Paulsen F. (2008). Dermal subsitute with the collagen-elastin matrix Matriderm in burn injuries: A comprehensive review. Handchir. Mikrochir. Plast. Chir. Organ Dtsch. Arb. Handchir. Organ Dtsch. Arb. Mikrochir. Peripher. Nerven Gefasse Organ V..

[199] Min J.H., Yun I.S., Lew D.H., Roh T.S., Lee W.J. (2014). The use of matriderm and autologous skin graft in the treatment of full thickness skin defects. Arch. Plast. Surg..

[200] Haslik W., Kamolz L.-P., Nathschläger G., Andel H., Meissl G., Frey M. (2007). First experiences with the collagen-elastin matrix Matriderm^®^ as a dermal substitute in severe burn injuries of the hand. Burns.

[201] Cervelli V., Brinci L., Spallone D., Tati E., Palla L., Lucarini L., De Angelis B. (2011). The use of MatriDerm^®^ and skin grafting in post-traumatic wounds. Int. Wound J..

[202] Nizioł M., Paleczny J., Junka A., Shavandi A., Dawiec-Liśniewska A., Podstawczyk D. (2021). 3D Printing of Thermoresponsive Hydrogel Laden with an Antimicrobial Agent towards Wound Healing Applications. Bioengineering.

[203] De Vries H., Zeegelaar J., Middelkoop E., Gijsbers G., Van Marle J., Wildevuur C., Westerhof W. (1995). Reduced wound contraction and scar formation in punch biopsy wounds. Native collagen dermal substitutes. A clinical study. Br. J. Dermatol..

[204] Cheshire P.A., Herson M.R., Cleland H., Akbarzadeh S. (2016). Artificial dermal templates: A comparative study of NovoSorb™ biodegradable temporising matrix (BTM) and Integra^®^ dermal regeneration template (DRT). Burns.

[205] Schneider J., Biedermann T., Widmer D., Montano I., Meuli M., Reichmann E., Schiestl C. (2009). Matriderm^®^ versus Integra^®^: A comparative experimental study. Burns.

[206] Shahrokhi S., Arno A., Jeschke M.G. (2014). The use of dermal substitutes in burn surgery: Acute phase. Wound Repair Regen..

[207] Zhai M., Yoshii F., Kume T., Hashim K. (2002). Syntheses of PVA/starch grafted hydrogels by irradiation. Carbohydr. Polym..

[208] Khil M.S., Cha D.I., Kim H.Y., Kim I.S., Bhattarai N. (2003). Electrospun nanofibrous polyurethane membrane as wound dressing. J. Biomed. Mater. Res. Part B Appl. Biomater. Off. J. Soc. Biomater. Jpn. Soc. Biomater. Aust. Soc. Biomater. Korean Soc. Biomater..

[209] Liu L., Li X., Nagao M., Elias A.L., Narain R., Chung H.-J. (2017). A pH-Indicating colorimetric tough hydrogel patch towards applications in a substrate for smart wound dressings. Polymers.

[210] Navarro J., Din M., Janes M.E., Swayambunathan J., Fisher J.P., Dreher M.L. (2019). Effect of print orientation on microstructural features and mechanical properties of 3D porous structures printed with continuous digital light processing. Rapid Prototyp. J..

[211] Milojević M., Harih G., Vihar B., Vajda J., Gradišnik L., Zidarič T., Stana Kleinschek K., Maver U., Maver T. (2021). Hybrid 3D printing of advanced hydrogel-based wound dressings with tailorable properties. Pharmaceutics.

[212] Danielsson P., Fredriksson C., Huss F. (2009). A novel concept for treating large necrotizing fasciitis wounds with bilayer dermal matrix, split-thickness skin grafts, and negative pressure wound therapy. Wounds (King of Prussia, Pa.).

[213] Wagstaff M.J., Salna I.M., Caplash Y., Greenwood J.E. (2019). Biodegradable Temporising Matrix (BTM) for the reconstruction of defects following serial debridement for necrotising fasciitis: A case series. Burns Open.

[214] Damkat-Thomas L., Greenwood J.E., Wagstaff M.J. (2019). A synthetic biodegradable temporising matrix in degloving lower extremity trauma reconstruction: A case report. Plast. Reconstr. Surg. Glob. Open.

[215] Li S., Chen N., Li X., Li Y., Xie Z., Ma Z., Zhao J., Hou X., Yuan X. (2020). Bioinspired double-dynamic-bond crosslinked bioadhesive enables post-wound closure care. Adv. Funct. Mater..

[216] Auger F.A., Lacroix D., Germain L. (2009). Skin substitutes and wound healing. Ski. Pharmacol. Physiol..

[217] Liu X., Liu Y., Du J., Li X., Yu J., Ding B. (2021). Breathable, stretchable and adhesive nanofibrous hydrogels as wound dressing materials. Eng. Regen..

[218] Hoare T.R., Kohane D.S. (2008). Hydrogels in drug delivery: Progress and challenges. Polymer.

[219] Bacelar A.H., Cengiz I.F., Silva-Correia J., Sousa R.A., Oliveira J.M., Reisa R.L. (2017). Smart” hydrogels in tissue engineering and regenerative medicine applications. Handb. Intell. Scaffolds Tissue Eng. Regen. Med..

[220] Badawy A.R., Hassan M.U., Elsherif M., Ahmed Z., Yetisen A.K., Butt H. (2018). Contact lenses for color blindness. Adv. Health Mater..

[221] He Y., Wang F., Wang X., Zhang J., Wang D., Huang X. (2021). A photocurable hybrid chitosan/acrylamide bioink for DLP based 3D bioprinting. Mater. Des..

[222] Robles-Martinez P., Xu X., Trenfield S.J., Awad A., Goyanes A., Telford R., Basit A.W., Gaisford S. (2019). 3D printing of a multi-layered polypill containing six drugs using a novel stereolithographic method. Pharmaceutics.

[223] Cereceres S., Lan Z., Bryan L., Whitely M., Wilems T., Greer H., Alexander E.R., Taylor R.J., Bernstein L., Cohen N. (2019). Bactericidal activity of 3D-printed hydrogel dressing loaded with gallium maltolate. APL Bioeng..

[224] Song C.K., Kim M.-K., Lee J., Davaa E., Baskaran R., Yang S.-G. (2019). Dopa-empowered Schiff base forming alginate hydrogel glue for rapid hemostatic control. Macromol. Res..

[225] Süpple J., von Glasenapp J., Hofmann E., Jost-Brinkmann P.-G., Koch P.J. (2021). Accurate bracket placement with an indirect bonding method using digitally designed transfer models printed in different orientations—An in vitro study. J. Clin. Med..

[226] Mueller J., Shea K. The effect of build orientation on the mechanical properties in inkjet 3D printing. Proceedings of the 2015 International Solid Freeform Fabrication Symposium.

[227] Jaiswal L., Shankar S., Rhim J.-W. (2019). Carrageenan-based functional hydrogel film reinforced with sulfur nanoparticles and grapefruit seed extract for wound healing application. Carbohydr. Polym..

